# Considering the Cellular Composition of Olfactory Ensheathing Cell Transplants for Spinal Cord Injury Repair: A Review of the Literature

**DOI:** 10.3389/fncel.2021.781489

**Published:** 2021-11-17

**Authors:** Mahjabeen Miah, Patrizia Ferretti, David Choi

**Affiliations:** ^1^Spinal Repair Unit, Department of Brain Repair and Rehabilitation, UCL Queen Square Institute of Neurology, London, United Kingdom; ^2^Developmental Biology and Cancer Department, UCL Great Ormond Street Institute of Child Health, London, United Kingdom

**Keywords:** cell transplantation, glial cells, olfactory ensheathing cells, cellular composition, neuroregeneration, spinal cord injury

## Abstract

Olfactory ensheathing cells (OECs) are specialized glia cells of the olfactory system that support the continual regeneration of olfactory neurons throughout adulthood. Owing to their pro-regenerative properties, OECs have been transplanted in animal models of spinal cord injuries (SCI) and trialed in clinical studies on SCI patients. Although these studies have provided convincing evidence to support the continued development of OEC transplantation as a treatment option for the repair of SCI, discrepancies in the reported outcome has shown that OEC transplantation requires further improvement. Much of the variability in the reparative potential of OEC transplants is due to the variations in the cell composition of transplants between studies. As a result, the optimal cell preparation is currently a subject of debate. Here we review, the characterization as well as the effect of the cell composition of olfactory cell transplantation on therapeutic outcome in SCI. Firstly, we summarize and review the cell composition of olfactory cell preparations across the different species studied prior to transplantation. Since the purity of cells in olfactory transplants might affect the study outcome we also examine the effect of the proportions of OECs and the different cell types identified in the transplant on neuroregeneration. Finally, we consider the effect of the yield of cells on neuroregeneration by assessing the cell dose of transplants on therapeutic outcome.

## Introduction

**FIGURE 1:**
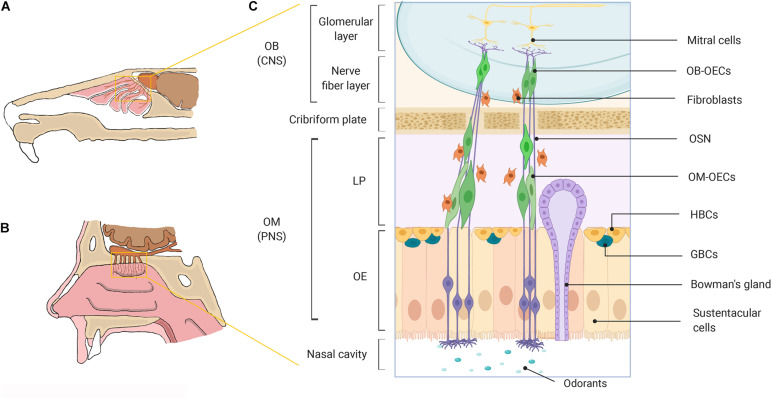
A schematic representation of the olfactory system in mammals. **(A)** Rodent nasal cavity. **(B)** Human nasal cavity. **(C)** A detailed illustration of the major cell constituents of the mammalian olfactory system. OECs can be obtained from the OM lining the nasal cavity located within the PNS and from the nerve fiber layer of the OB, located within the cranial cavity in the CNS. OB, olfactory bulb; OM, olfactory mucosa; CNS, central nervous system; PNS, peripheral nervous system; LP, lamina propria; OE, olfactory epithelium; OECs, olfactory ensheathing cells; OSN, olfactory sensory neuron; HBCs, horizontal basal cells; GBCs, globose basal cells. Created with BioRender.

Neurogenesis in the mammalian olfactory system has been well established through early *in vitro* and *in vivo* studies in rodents and other species (Graziadei and Graziadei, 1979a; Graziadei and Okano, 1979c; Graziadei et al., 1980; Schwob et al., 1995; Kornack and Rakic, 2001; Hahn et al., 2005). Neurogenesis occurs in the olfactory epithelia (OE) where short lived olfactory sensory neurons (OSNs) are replaced in order to preserve olfactory function throughout life, though despite ongoing neurogenesis, neuronal regeneration has been shown to decline with advancing age (Kondo et al., 2010; Holbrook et al., 2011; Mobley et al., 2014). The continual regeneration of OSNs either during normal cell turnover or after injury to the olfactory nerve is maintained by multipotent and/or neuronal precursor cells (globose basal cells, GBCs and horizontal basal cells, HBCs) in the basal layer of the OE (Graziadei and Graziadei, 1979b; Mackay-Sim and Kittel, 1991; Leung et al., 2007; Holbrook et al., 2011; Chen C. R. et al., 2014; Schwob et al., 2017; Figure 1). Regeneration of OSNs is thought to be highly dependent on specialized glial cells called olfactory ensheathing cells (OECs) which, exhibit unique neurotrophic properties. OECs play a vital role in supporting and guiding newly formed olfactory sensory axons from their origin in the peripheral nervous system (PNS) environment of the olfactory mucosa (OM) to establish synaptic connections with their targets in the normally non-permissive central nervous system (CNS) environment of the olfactory bulb (OB) to facilitate odor detection (Raisman and Li, 2007; Roet and Verhaagen, 2014). In the olfactory nerve OECs provide structural support by ensheathing large bundles of non-myelinated olfactory sensory axons to form a conduit for newly generated axons to grow through as they extend all the way from the lamina propria (LP) toward the nerve fiber layer of the OB (Doucette, 1990, 1991; Tennent and Chuah, 1996; Field et al., 2003; Raisman and Li, 2007; Choi et al., 2008a). Olfactory ensheathing cells further support neural regeneration by secreting a range of neurotrophic and guidance factors (Woodhall et al., 2001; Lipson et al., 2003; Chung et al., 2004), phagocytosing axonal debris and/or invading pathogens (Leung et al., 2008; Panni et al., 2013; Su et al., 2013; Nazareth et al., 2015) and integrating and interacting with other cell types such as CNS glia and astrocytes (Chuah et al., 2011). Owing to the strong pro-regenerative properties of OECs, as well as their unique migratory properties (Tennent and Chuah, 1996; Ekberg et al., 2012), OECs have been investigated for transplant-mediated repair of CNS injury with promising but highly variable outcomes.

Transplantation of OECs in experimental animal models of spinal cord injuries (SCI) has been extensively studied and together with studies in humans have provided convincing evidence to support the continued development of OEC transplantation as a treatment option (Feron et al., 2005; Mackay-Sim et al., 2008; Granger et al., 2012; Tabakow et al., 2013, 2014; Watzlawick et al., 2016; Nakhjavan-Shahraki et al., 2018). Transplantation of OECs in animal studies has been shown to have a wide range of therapeutic effects by promoting axonal regeneration, restoring function, reducing cavity and scar formation, remyelinating axons (a function they do not serve in the nasal cavity), modulating neuroinflammation and promoting neuronal survival and plasticity (Li et al., 1997, 2003; Ramon-Cueto et al., 1998, 2000; Lu et al., 2002; Plant et al., 2003; Ramer et al., 2004; Lopez-Vales et al., 2006; Lankford et al., 2008). A systematic meta-analysis of 62 experimental studies in rodent models of SCI found OEC transplants improved functional recovery with an effect size of 19.2%, after correcting for publication bias and missing data (Watzlawick et al., 2016). The efficacy of OEC transplants was further corroborated in a recent systematic meta-analysis of 933 animals (control group = 464 and treatment group = 469), which confirmed that OEC transplantation significantly improves motor function recovery in animals after SCI (Nakhjavan-Shahraki et al., 2018). However, despite the successes reported in published studies of regeneration within the CNS, a number of studies have been unable to confirm these positive results (Takami et al., 2002; Deumens et al., 2006; Lu et al., 2006; Steward et al., 2006). Conflicting evidence in reported outcomes has led to doubts about the efficacy of OECs for CNS repair.

The variation in the reparative potential of OEC transplants is not fully understood and may be due to several factors including differences in SCI model, cell delivery techniques, adequate assessment of functional outcome measures and perhaps most importantly the considerable differences in the cell purity and yield of cell transplants between studies. OEC cultures generated *in vitro* from the olfactory region are derived from either the OM or OB and typically contain a heterogeneous mix of cell types including olfactory nerve fibroblasts (ONFs) and other accessory cells, along with OECs (Lindsay et al., 2010; Chen L. et al., 2014; Yao et al., 2018). The yield of OECs, often measured as percentage proportion differs between the two olfactory regions, with higher yields being generated *in vitro* from OB biopsies than OM biopsies (Jani and Raisman, 2004; Kueh et al., 2011; Mayeur et al., 2013; Ibrahim et al., 2014). However, for clinical application, autologous OM transplants are the preferred source as mucosal biopsies are easily accessible via less invasive intranasal endoscopy, avoiding the requirement for intrusive intracranial surgery to harvest biopsies from the OB (Choi et al., 2008b; Andrews et al., 2016; Holbrook et al., 2016). In addition to the anatomical source, the proportions and types of cells present in olfactory transplants is greatly influenced by the *in vitro* methods/conditions used to isolate and culture OEC preparations (Kawaja et al., 2009; Ekberg and St John, 2014; Reshamwala et al., 2020). As there is a lack of consensus with regards to the optimal cell culture protocol for OECs, it is likely that variations in cell preparations lead to variability in treatment outcomes.

The optimal cell preparation is still a subject of debate. OEC purification methods from the bulb and mucosa have been developed to separate OECs from the many potentially “contaminating” cells (Kawaja et al., 2009). Although, a number of groups have reported beneficial effects by transplanting purified OECs (Ramon-Cueto and Nieto-Sampedro, 1994; Toft et al., 2013), several studies have demonstrated unpurified, mixed cell preparations of OECs and other cells from the olfactory niche might be important for neural repair. Raisman et al. have argued that ONFs are a crucial component of OEC transplants in facilitating the formation of organized OEC bridges through which axons can regenerate across injured tissue. Unpurified primary OB cultures containing 50:50 ratio of OECs and ONFs were suggested as the optimal proportion of cell types needed for neural repair (Li et al., 1998, 2003; Keyvan-Fouladi et al., 2003). The reported benefits of additional cells in olfactory cell transplants based on rodent studies has led to the transplantation of unpurified mixed cell cultures in human studies, with varying degrees of neurological and neurophysiological improvement being observed (Tabakow et al., 2013, 2014). While, studies in humans have provided vital data about the safety and feasibility of olfactory cell transplantation (Yao et al., 2018) clinical application of OEC transplants for human transplantation, will require an understanding of what constitutes the human cell cultures and the importance of additional cell types in the cultures for SCI repair.

Cell based therapies have been a major focus in the development of new treatments for SCI. While numerous cell types have been investigated, OEC transplantation remains among the most widely studied and has emerged as a promising treatment for SCI repair (Tetzlaff et al., 2011; Assinck et al., 2017; Willison et al., 2020). However, given the variability in outcomes, the therapy still requires improvement. To help guide the development of OEC transplantation for SCI repair, the objective of the present review was to summarize and review the current literature pertaining to the cellular characteristics and composition of OEC cell preparations for transplantation in the treatment of SCI across the different species studied. Specifically, with a focus on the effect of three main parameters on neuroregeneration and repair in traumatic SCI: proportion of OECs in cell preparations, proportion of additional cells in the transplant (namely, fibroblasts) and total cell number transplanted. These are important factors that must be optimized in the clinical translation of OEC transplants for human SCI.

## Methods

### Search Strategy

A literature search was conducted using PubMed database for original studies related to the therapeutic application of olfactory bulb and mucosa cell preparations in traumatic models of SCI that were published from January 1990 up until 11th April 2021. The search terms are reported in Table 1. The titles and abstracts, and when not explicit full texts were screened to select studies in SCI models. The methods and results sections of these publications were read to identify studies that met the pre-defined inclusion criteria. Only studies that measured the proportions of OECs and/or mentioned the presence of additional cell types in bulb and mucosal cultures in SCI repair were included. Studies that included the application of unpurified (primary) and purified OEC cultures in combined treatments (e.g., with cells, scaffolds, and drugs, etc.) to measure synergistic effects were also included, as long as the proportions of cells in olfactory tissue culture were reported. All the studies included in the analysis of this review are summarized in Supplementary Table S1. Studies that mentioned the presence of OECs and its cell constituents but did not measure the proportions of these cells were excluded along with studies that transplanted complete non-dissociated pieces olfactory tissue. Studies that evaluated the therapeutic application of bulb and mucosal OECs and other cell types exclusively in *in vitro* models and non-traumatic models of SCI were excluded. Reviews and case reports were also excluded.

**TABLE 1:** PubMed search.

(“olfactory mucosa”[MeSH Terms] OR (“olfactory”[All Fields] AND “mucosa”[All Fields]) OR “olfactory mucosa”[All Fields]) OR (“olfactory bulb”[MeSH Terms] OR (“olfactory”[All Fields] AND “bulb”[All Fields]) OR “olfactory bulb”[All Fields])	AND	(“olfactory ensheathing cells”[All Fields] OR “OEC”[All Fields] OR (“olfactory”[All Fields] AND “ensheathing”[All Fields] AND “cells”[All Fields]) OR “olfactory ensheathing glia”[All Fields] OR “OEG”[All Fields] OR (“olfactory”[All Fields] AND “ensheathing”[All Fields] AND “glia”[All Fields]))	AND	(“1990/01/01”[PDAT]: “3000/12/31”[PDAT])

### Data Extraction

The data from those studies that fitted the criteria were extracted into a table format to include the following information: (1) reference details (PubMed ID), (2) OEC culture source (donor species and olfactory tissue source), (3) recipient species, (4) injury model, (5) the total number of cells transplanted, (6) characterization antibodies used to identify the cells in culture, (7) cell purification methods (if applied), (8) purity of the final cell preparation, i.e., proportions of OECs in percentage and where mentioned additional cells, (9) method to determine purity, (10) the reported structural/functional outcome measures such as, locomotor recovery, axonal regeneration, remyelination, tissue sparing, and sensory preservation etc. The studies were identified either as positive or negative studies based on the resulting outcome measure following the application of OM and OB cell culture transplants. Positive studies were identified on the basis of any reported improvements in any measure of functional or histological outcome and negative studies were identified by the lack of therapeutic effect after transplantation. Where a single publication included more than one experiment in relation to, i.e., OEC source, injury and purification methods, the data was extracted and treated as an independent experiment. For the analysis of cellular composition, where a percentage range of values for OEC and other cell types was given, the midpoint of the range was selected for analysis. Where the composition was marked as >X%, the X% value was selected for analysis; where the compositions were given as separate percentage values within the same experiment, these were treated as separate experiments.

## Results and Discussion

### Study Details

**FIGURE 2:**
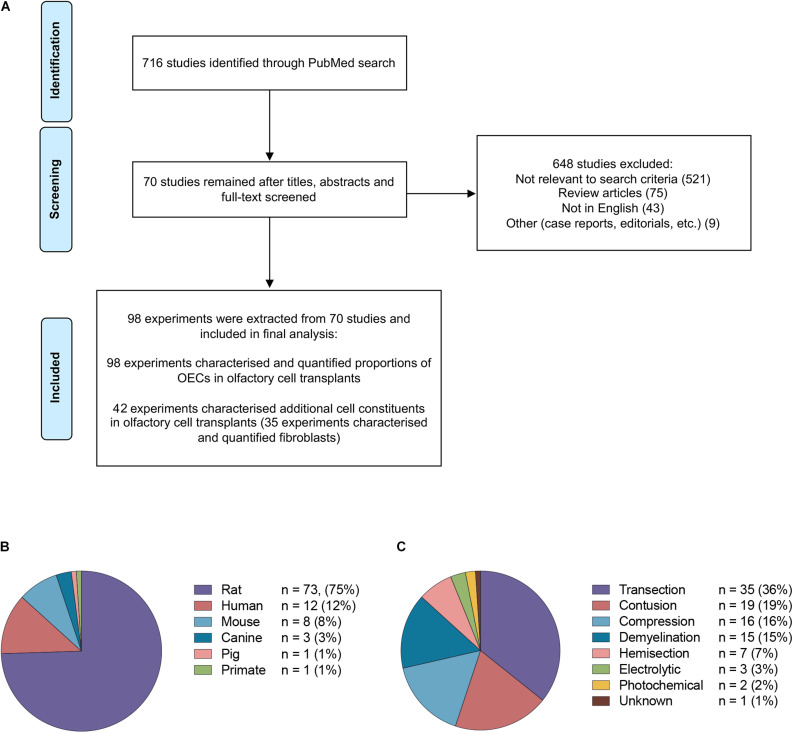
Details of studies included in the analysis. **(A)** Flow diagram of the study selection process. A total of 70 studies were identified for systematic review. A total of 98 experiments were extracted from the 70 studies based on the inclusion criteria. **(B)** The species of olfactory cell transplants obtained for transplantation in the studies included in the review (*n* = no. of experiments). **(C)** The types of injury models investigated for transplantation in the studies included in the review (*n* = no. of experiments).

The study selection process is summarized in Figure 2A. The PubMed search strategy yielded a total of 716 studies using the search terms outlined. Of the studies that were identified, 648 were excluded based on either title, abstract or after full text screening. Ultimately, 70 publications matched the search criteria and were included for the final analysis. When the same study compared different experimental groups in relation to OEC transplant source, OEC proportions, injury and purification methods these were treated as separate experiments. Consequently, a total of 98 experiments were extracted from 70 publications.

The majority of the data collected from these studies related to rodent olfactory derived cultures (*n* = 82, 83.7%). In comparison, the composition of cells in human OEC cultures (*n* = 12, 12.2%) and large animal model OEC cultures (*n* = 5, 5.1%) were reported less often (Figure 2B). Seventy-eight (79.6%) experiments used allogeneic transplants, 13 used xenogeneic (13.3%) and the remaining studies applied autologous transplantation (*n* = 7, 7.1%). The type of spinal cord injury in published studies varied considerably (Figure 2C). The most common injury models in the studies were transection in 35 (35.7%) experiments, contusion in 19 (19.4%), compression in 16 (16.3%), demyelination in 15 (15.3%), hemisection in 7 (7.1%), electrolytic in 3 (3.0%) and photochemical in 2 (2.0%) experiments, and in one study it was unclear which SCI model was applied (Zheng et al., 2017). It should be noted that in addition to the heterogeneity in injuries amongst the studies, there was also considerable heterogeneity with regards to the time of application of cells post injury, injection volumes, concentration of cells, number and localization of injections. The impact of these factors in OEC transplantation will not be discussed here as it has been reviewed elsewhere (Watzlawick et al., 2016; Nakhjavan-Shahraki et al., 2018). All but one study (Tabakow et al., 2014) in the review included a control or a comparison group.

### Composition of Olfactory Cell Transplants

To understand the influence of the types of cells present in the olfactory transplant in addition to OECs, we reviewed experiments reporting the characterization of the cell composition prior to transplantation. The characterization and quantification of cells in olfactory transplants was mostly limited to OECs (*n* = 98, 100%). Additional cells in the transplant were described less often (*n* = 42, 42.9%), with fibroblasts/meningeal cells (*n* = 35, 35.7%) being characterized most often. Despite the complexity of the cell mixture, overall we found that OEC transplantation studies did not focus on characterizing the additional cell types in the cell transplant, (*n* = 19, 19.4%), summarized in Supplementary Table S2. The characterization and quantification of additional cell constituents in transplants was mostly limited to OB transplants with the majority of studies describing rat derived cell preparations (*n* = 15), and included two studies describing canine and two studies describing human OEC cell transplants. The additional cell types identified in these transplants included astrocytes (*n* = 6), endothelial cells (*n* = 3), macrophages/microglia (*n* = 2), oligodendrocyte progenitors (*n* = 2), oligodendrocyte precursors (*n* = 1), Schwann cells (*n* = 3) and stem cells/neuronal cells/connective tissue (*n* = 1). It is generally accepted that OM-OEC transplants will contain several cell types compared to OB-OEC transplants (Lindsay et al., 2010; Holbrook et al., 2011, 2016; Chen C. R. et al., 2014; Borgmann-Winter et al., 2015), but despite this only 3 out of 19 studies discussed the presence of accompanying cells in OM transplants, of which the characterization was very limited. Granger et al. (2012) found that cells in addition to OECs and fibroblasts made up ∼2% of the transplant in canine unpurified OM cell transplants however, they did not immunocharacterize these cells. Others (Kalincik et al., 2010b; Stamegna et al., 2011) described using HNK-1 marker to label myelinating purified rat Schwann cell transplants. Both studies reported rat OM-OEC cell transplants were negative for HNK-1 but speculated the possibility of non-myelinating Schwann cell contamination, which lacks or downregulates the HNK-1 epitope *in vitro*.

None of the studies included in the review discussed the influence of additional cells in SCI repair, other than fibroblasts (discussed in section “Proportions of Fibroblasts in the Mixed Olfactory Cell Transplants”) and coupled with a lack of sufficient number of studies characterizing and quantifying the proportions of different cell types within the OEC cell transplant it was not possible to assess the impact of additional cells in olfactory cell transplants. It is possible that the different cellular components of olfactory transplants may not have been reported as the majority of studies transplanted highly purified preparations containing > 70% OECs, *n* = 67 (68.4%), of which 48 (71.6%) of experiments contained >90% OECs. However, some studies found that even in highly purified transplants additional cells remained, albeit in small amounts. To date, a number of methods have been developed to identify and purify heterogeneous cultures to obtain highly purified OEC cultures. Such methods include but are not limited to differential adhesion, immunopanning, FACS and selective media [reviewed elsewhere (Kawaja et al., 2009)]. Due to the paucity of data it is not known how purification methods might affect the therapeutic efficacy of OECs and the cell constituents of OEC cultures. One study included in the analysis of the review investigated the influence of the cell culture preparation of purified OECs prior to transplantation and found that the method of cell purification and the age of cells in culture have influence on the growth-promoting and neuroprotective effects of OECs after transplantation into the injured spinal cord (Novikova et al., 2011). There are almost as many descriptions of OEC preparations as there are laboratories exploring their biology. The purification method and length of culture are likely to be of importance and further research is required to identify the methods for preparing cells in culture prior to their implantation that yield the best outcome for SCI repair. The lack of phenotypic and functional characterization of the subpopulations of cells of OEC cultures across the studies reviewed are also likely due to a general lack of specific *in vitro* markers making the identification and quantification of the additional cell types more challenging (Yao et al., 2018). The most commonly used marker to identify OECs, p75 neurotrophin receptor is also expressed *in vitro* by Schwann cells (Chen C. R. et al., 2014), astrocytes (Coutts et al., 2013) and lamina propria mesenchymal stem cells (MSCs) (Lindsay et al., 2013, 2017) which may be present in OM derived cell transplants under certain conditions. Additionally, Thy1.1 marker was not only used to characterize fibroblasts (Lankford et al., 2008; Toft et al., 2013; Ibrahim et al., 2014), but was also used to characterize stem cells, neuronal cells and connective tissue (Toft et al., 2007). Of the studies identifying additional cells in the transplants the cell types described varied greatly in characterization and proportions between studies, these differences are likely due to be influenced by several factors including (1) source and site of biopsy tissue (either bulbar or mucosal), (2) interspecies and intraspecies (age, bodyweight and sex of animals) differences, (3) cell culture conditions – time in culture, reagents, passage number, purification techniques applied, (4) the storage of olfactory tissue prior to *in vitro* cell culture, (5) the marker(s) used to identify cell types. To develop the optimal olfactory cell transplant will require an understanding of the influence of each of these factors.

### Proportions of Olfactory Ensheathing Cells in Olfactory Cell Transplants

**FIGURE 3:**
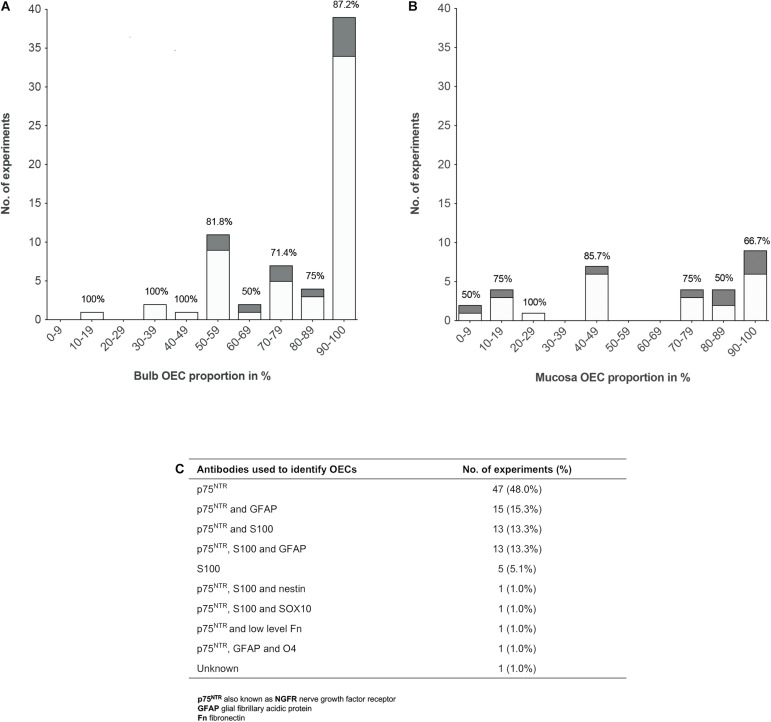
Comparison of the impact of OEC proportions in olfactory cell transplants on therapeutic outcome. **(A)** Comparing the% proportion of olfactory bulb derived OECs on therapeutic outcome. Each bar is labeled with the% of positive outcome for each condition (*n* = 67 experiments). **(B)** Comparing the% proportion of olfactory mucosa derived OECs on therapeutic outcome. Each bar is labeled with the% of positive outcome for each condition (*n* = 31 experiments). **(C)** Immunocytochemical makers used in the characterization of OECs in the studies included in the review (*n* = 98 experiments). The white bars represent the positive outcome experiments and the gray bars represent the negative outcome experiments.

To analyze the proportion of OECs in olfactory cell transplants in SCI, data from 98 experiments were extracted from the 70 studies analyzed (Figure 3). All studies identified cells through immunocytochemical staining. One study quantified the percentage proportions of cells through flow cytometry, while the remaining studies performed manual cell counts of positively stained cells from micrograph images. The majority of experiments (92, 93.9%) used p75^NTR^ either on its own or in combination with other markers to identify OECs. The antibody markers used to identify OECs are summarized in Figure 3C.

The results demonstrated a tendency for transplanting OB derived cell preparations (*n* = 67, 68.4%) over OM derived cell preparations (*n* = 31, 31.6%). Overall, the data showed that more experiments on transplantation of OB and OM in SCI model reported therapeutic benefits (*n* = 56, 83.6% and *n* = 22, 71.0%, respectively) than an absence of beneficial effects (*n* = 11, 16.4% and *n* = 9, 29.0%, respectively). What is striking in Figure 3A is the general preference for transplanting highly pure cell preparations containing > 90% proportion of OB-OECs (*n* = 39, 39.8%) compared to mixed cell transplants. Over 86% of these studies reported neurological improvement after transplantation, which included axonal remyelination, axonal regeneration, electrophysiological and functional recovery and reduction of glial scar formation amongst other improvements. One study included in the analysis compared the effect of varying proportions of primary (unpurified) OB and OM transplants (containing 70 and 15% OECs, respectively) and purified OB and OM transplants (containing 97 and 98% OECs, respectively) with a medium only transplant control group in a complete transection injury model in rats (Mayeur et al., 2013). This study demonstrated that while all olfactory treated groups were equally capable of inducing electrophysiological improvement, reducing astrocytic reactivity in the lesion and glial scar formation, only primary OB and purified OB and purified OM transplants were capable of promoting axonal regrowth and inducing functional recovery compared to the control group. The absence of neural repair when transplanting primary OM transplants was reported to be due to low yields of OECs and/or inhibition of repair by contaminating cells present in the primary OM (Mayeur et al., 2013). Similarly, another study reported pure preparations of OB-OECs (containing > 97% OECs) provided optimal regenerative and integrative properties into the transected rat spinal cord compared to unpurified mixed OM cell transplants (containing 12% OECs) and mixed OB cell transplants (containing 50% purified OB-OECs and 50% purified OB-fibroblasts, both populations were purified separately and combined in a mixed suspension for transplantation). Although unpurified OM-OECs and mixed OB-OECs/OB-fibroblasts transplants were capable of supporting axonal regeneration and preventing cavity formation within the lesion, these mixed cell transplants also resulted in longer lesion lengths, greater astrocytic reaction and extensive scarring. The differences in host transplant reactivity were confirmed by transplanting the cell preparations into the normal, non-lesioned spinal cord (Toft et al., 2013). Overall, the results might indicate that higher proportions of OECs in the transplant lead to greater neurological improvement. However, not all studies reported therapeutic improvement after transplanting highly purified olfactory cell preparations. Both Barakat et al. (2005) and Pearse et al. (2007) reported limited survival of OECs, limited axonal regeneration and limited functional recovery in a contusion injury model in rats after transplanting OB cell preparations containing > 94% OECs transplanted directly into the lesion site. Pearse et al. (2007) suggested the absence of recovery was due to the poor survival of highly purified OEC transplants within the harsh environment of the injury site, as only ∼3% of OECs survived at 3 weeks after transplantation. In contrast, a 3 fold increase in survival of OECs was observed, when purified OECs were co-transplanted in the injury site in a 1:1 ratio with purified Schwann cells, which resulted in modest but significant locomotor recovery compared to injury-only control animals (Pearse et al., 2007). Likewise, Lakatos et al. (2003) found poor remyelination of demyelinated axons using highly purified transplants containing ∼94% OB-OECs. An estimated 3 fold increase in the degree of remyelination was observed when unpurified OEC preparations containing ∼69% OB-OECs, were transplanted in a CNS demyelination injury model in rats, compared to that achieved with purified OECs alone (discussed in further detail below). Additionally, in a single-blinded phase I human clinical trial using a matched group of injury-only controls, Feron et al. (2005) tested the safety and feasibility of autologous OEC transplants in the injured spinal cord. Three patients with chronic thoracic SCI, characterized as American Spinal Injury Association (ASIA) category A on the Impairment Scale, were injected with high proportions of OM-OECs (containing > 80% OECs). At 3 years after transplantation no significant functional recovery was observed except in one patient who showed an improvement in light touch and pin prick sensitivity (Mackay-Sim et al., 2008). The study confirmed the safety and feasibility of the procedure as no adverse events occurred post-transplantation.

Although, the data reveals a preference for transplanting highly purified populations of both bulb and mucosa derived OECs rather than mixed cell preparations, both Figures 3A,B show successful transplant-mediated functional repair was possible with low proportions of OECs in mixed cell transplants. Notably, the data collected shows mixed cell transplants containing < 50% OECs may be equally capable of promoting neurological improvement as highly purified transplants containing > 90% OECs. Both of these transplant groups showed that 83% of studies (24 out of 29 studies) using transplants containing < 50% OECs, as well as 83% of studies (40 out of 48 studies) using transplants containing > 90% OECs resulted in favorable outcomes. Unpurified mucosal cell transplants containing OEC proportions as low as 5% were capable of restoring paw reaching function in 5 out of 6 rats in a CST lesion model, in comparison to 8 lesioned, non-transplanted rats, which showed no functional recovery for up to 18 weeks after the lesion (Yamamoto et al., 2009). A study by Granger et al. (2012) also demonstrated that autologous OM cell transplants containing low proportions of OECs (∼10% OECs) were equally capable of improving locomotor function in companion dogs with chronic clinical SCI, as those receiving transplants containing higher proportions of OECs (∼80% OECs, proportions extrapolated from Supplementary Data). Additionally, a Phase I non-randomized controlled clinical trial using unpurified suspensions, described as a mix of autologous OECs and ONFs from the OM was transplanted in three patients with chronic thoracic SCI and compared to three unoperated, injured control patients (all patients graded ASIA A) (Tabakow et al., 2013). One year after transplantation functional improvement was observed in 2 out of the 3 patients receiving transplants with lower proportions of OECs, 10 and 12% OECs, which resulted in conversion from ASIA grade A to ASIA C and ASIA B, respectively. The third patient transplanted with a higher proportion of OECs, 25.7% OECs, remained at ASIA A but showed improved motor and sensory function of the first spinal segments below the injury. No improvements were observed in the control group. Following on from this study, the same group transplanted unpurified suspensions, described as autologous bulbar OEC/ONFs containing OEC proportions as low as 16% in a single patient with chronic thoracic transection injury (Tabakow et al., 2014) – similar injury to that of patient denoted T1 in the author’s 2013 study (Tabakow et al., 2013). Considerable improvements were observed in the patient’s sensory and motor function, as the patient converted from ASIA A to ASIA C within 11 months after transplantation. However, in contrast to the 2013 study (Tabakow et al., 2013) there were several differences in the study setting that may have contributed to the neurological recovery observed in the patient. OB cells were transplanted in a combinatorial treatment that included a peripheral nerve graft taken from the patient’s sural nerve, removal of inhibitory scar tissue in the lesion site and an intensive rehabilitation schedule. Additionally, others have noted, following surgery there appeared to be a possible decompression of a cyst which may have contributed to the recovery observed, together with the untethering of dural adhesions (Guest and Dietrich, 2015).

We were unable to find evidence for an optimal proportion of OECs that leads to neurological recovery. However, looking at the trends in the data collected successful transplant-mediated repair appeared to be possible using both low and high proportions of OECs in olfactory transplants. The studies reviewed also indicate that the proportions of OECs within the transplant cell population might not be linearly related to the neurological improvements observed. It is possible that instead a threshold proportion of OECs exists, which might be quite low that leads to repair. Nevertheless, while some studies suggest low proportions of OECs in mixed cell transplants are capable of promoting neurological recovery in both animals and humans, more studies are required to confirm the efficacy of such cell preparations, as a clear study bias toward testing cultures with high proportions of OECs makes it difficult to draw conclusions about the efficacy of cultures with lower OEC proportions. It is also possible that cells other than OECs present within the mixed cell transplant may have a beneficial effect on repair (discussed in more detail below). Clinical studies have shown that human OEC cell transplants are well tolerated in the spine with neurological improvements being observed in some patients. We identified 4 clinical studies that identified and quantified the proportions of OECs in the cell preparation prior to transplantation. These studies mostly transplanted autologous cell transplants (Feron et al., 2005; Tabakow et al., 2013, 2014) with one using allogenic cell transplants (Chen L. et al., 2014). While human clinical studies have demonstrated the safety and feasibility of OEC transplantation, investigations reporting the composition of OEC transplants are limited to a handful of studies with various proportions and yields being transplanted. Further investigation is required to determine the cell type and proportions that lead to repair, once this is identified we can develop clinically relevant methods for OEC cell transplants.

### Proportions of Fibroblasts in the Mixed Olfactory Cell Transplants

**FIGURE 4:**
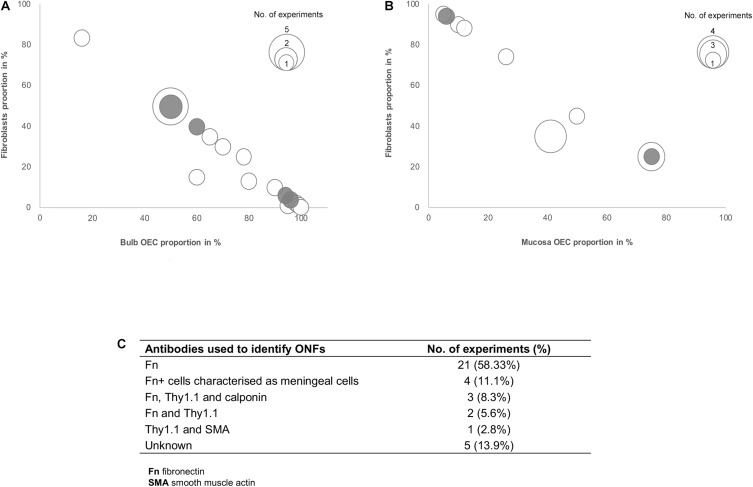
Comparison of the impact of the ratio of OECs to fibroblast proportion in olfactory cell transplants on therapeutic outcome. **(A)** Comparison of the% proportion of olfactory bulb derived OECs/fibroblast ratio on therapeutic outcome (*n* = 21 experiments). **(B)** Comparison of the% proportion of olfactory mucosa derived OECs/fibroblast ratio on therapeutic outcome (*n* = 14 experiments). **(C)** Immunocytochemical markers used in the characterization of fibroblasts in the studies included in the review (*n* = 35 experiments). The white bars represent the positive outcome experiments and the gray bars represent the negative outcome experiments.

Several studies included in this review suggested that the effects of olfactory cell transplants might not solely depend on the OEC component of the transplant and additional cell populations within the mixed cell transplant may be of importance in supporting the reparative properties of olfactory transplants. Fibroblasts in the transplant cell population were most often described and different groups suggested different optimal proportions of the combination of OECs and fibroblasts in the transplant. To analyze the relationship between proportions of OECs and fibroblasts in olfactory cell transplants, data from 35 experiments were extracted from the 22 studies that characterized and quantified fibroblasts (Figure 4). All studies included in the analysis identified cells through immunocytochemical staining and quantified the percentage proportions of cells through manual cell counts of positively stained cells from micrograph images (Figure 4C). The majority of experiments used fibronectin either on its own or in combination with other markers to identify fibroblasts (*n* = 31, 88.6%), the characterization of OECs is described above. It should be noted that a study included in the analysis in Figure 4, described cells immunoreactive for fibronectin as meningeal cells, these cells were cultured from rat OB (Lakatos et al., 2003) and were often described by others as OB fibroblasts (Li et al., 2003; Andrews and Stelzner, 2004; Bretzner et al., 2008).

The data reviewed suggests there is no clear relationship between the proportions of fibroblasts in transplants and neurological improvements in the injured spine. The trend in the data shows cell preparations containing 50% OECs and 50% fibroblasts appear to be transplanted most often (*n* = 7, 20%). These studies are mostly positive, with 78% (*n* = 7) reporting neurological improvement after transplantation, indicating that fibroblasts might play a role in supporting the reparative properties of olfactory transplants. Studies by the Raisman group, consistently reported unpurified OB derived transplants which were characterized as containing a mix of 50% OECs and 50% fibroblasts, as optimal for the promotion of functional repair in rat SCI models (Keyvan-Fouladi et al., 2003; Li et al., 2003, 2004; Ibrahim et al., 2014). The mechanisms underlying the therapeutic effect of transplants containing 50% OECs with 50% ONFs was demonstrated to be necessary for the formation of a sheath similar to their physiology *in situ*, where ONFs form an outer perineurial-like wrapping around bundles of OECs ensheathing a highly organized tissue bridge consisting of an aligned array of nerve fibers (Li et al., 1998). Keyvan-Fouladi et al. (2003) reported unpurified OB transplants, when transplanted 2 months after partial injury to the corticospinal tract (CST) in rats, were capable of restoring directed forepaw retrieval, compared to non-transplanted (injury only) rats, which failed to show return of retrieval for up to 6 months after the injury. In support of the use of unpurified OB transplants, Ibrahim et al. (2014) concluded that superior reparative effects, such as axonal regeneration and forepaw grasping observed in a rhizotomy injury model in rats, are attributed to transplants containing equal proportions of OECs and fibroblasts in unpurified OB cell transplants. In contrast, unpurified OM transplants which were largely composed of fibroblasts and low proportions of OECs (∼95% fibroblasts and ∼5% OECs), failed to exert regenerative effects in the same injury model. However, other studies included in the analysis have challenged the claim that 50:50 mixture of OECs/fibroblasts in the transplant is the optimal composition for transplant-mediated repair of the injured spine and have instead suggested the regenerative properties of OECs are enhanced when OECs form the substantial majority of the cells in the transplant. Toft et al. (2013) reported transplantation of 50:50 mixture of OB-OECs/OB-fibroblasts (each population was purified separately and then combined for transplantation) resulted in unfavorable responses, such as longer lesion length, marked astrocytic response and extensive scarring compared to highly purified OB-OEC transplants, containing > 97% OECs (discussed above). Similarly, Lakatos et al. (2003) demonstrated cell transplants containing equal proportions of OB-OECs and OB-fibroblasts (each population was purified separately and then combined for transplantation) had poor remyelinating capacity in a CNS demyelination injury model in rats. Instead they found transplants containing 70% purified OB-OECs combined with 30% purified ONFs resulted in approximately 2.5 times greater remyelination in comparison. Histological examination revealed when ONFs were present as a component of unpurified OB transplants (containing ∼60% OECs) they adopted an arrangement similar to that described by Li et al. (1998) the fibroblasts loosely encircled bundles of OECs containing myelinated axons creating a bridge, while transplants containing 70% purified OB-OECs combined with 30% purified OB-fibroblasts largely resulted in compacted clumps or cords of cells. The formation of OEC bridges may not necessarily be a requirement for repair as remyelination was equally possible using both unpurified and purified transplants (70:30 OECs and fibroblasts). Additionally, remyelination using these preparations was approximately 3-fold greater compared to that achieved with purified OECs alone. Andrews and Stelzner (2007) also acknowledged the importance of including fibroblasts, suggesting that the inclusion of at least 20% fibroblasts in OEC transplants enhanced the survival of OECs within the lesion compared to the limited survival of highly purified Schwann cells in a dorsal column crush injury model in rats.

Most human OEC studies consisted of clinical investigations and literature regarding human OEC xenotransplants is fragmentary. We identified only three studies reporting results of human OEC in rat models of SCI. There were no reports of human OEC transplantation in large animal models of SCI. Collins et al. (2018) transplanted unpurified human OB-OECs containing 90% OECs, along with fibroblasts and MSCs (characterization and quantification not stated), embedded within a collagen scaffold in a transection injury model. They found that half of the treated group recovered proprioception while the other half did not. In their previous study (Ibrahim et al., 2009) using the same injury model but using rat OB-OEC transplants (containing 50% OECs and 50% fibroblasts) they found greater (70% of rats) recovery of proprioception and fewer (30% of rats) lacking recovery amongst the rats receiving treatment. It was suggested the partial functional recovery using human OB-OECs could be due to higher proportions of OECs and fewer fibroblasts in the OB transplant. They also found that transplanted human OB-OECs showed limited migration and were predominantly located at the lesion site, which was similarly described in another study using highly purified human OM-OECs transplanted into the spinal cord of immunodeficient rats (Deng et al., 2006). Deng et al. (2006) compared the proliferation, survival and migration of highly purified human and rat OM-OECs transplants containing 100% OECs in a hemisection injury model. They found both rat and human OM-OECs rapidly stopped dividing following implantation and showed similarly minimal survival and migration using highly purified transplants in injured animals compared to intact animals. Human OECs have also been shown to share similar repair properties to rat OECs in terms of remyelination. Barnett et al. (2000) demonstrated human OB-OECs containing 30–66% OECs (transplants also contained fibroblasts and endothelial cells, proportions were not stated), were capable of remyelinating demyelinated CNS axons in rat spinal cord. Additionally, they demonstrated that human OECs growth factor requirements were similar to those of rat OECs. Whilst human OECs might share many properties with their rat counterpart, further investigation is required to determine the *in vivo* repair properties of human OECs, which are still poorly defined.

The studies reviewed indicate that the regenerative properties of OECs are enhanced when they are co-transplanted with fibroblasts which have been reported to facilitate a favorable environment by promoting axonal regeneration, remyelination, survival of OECs and functional recovery after SCI. Furthermore, olfactory fibroblasts in the transplant have been shown to provide important growth factors and a permissive substrate that stimulates the proliferation of OECs (Yui et al., 2011). Looking at the trend in the data we were unable to infer a relationship between the ratio of OECs and fibroblasts in the transplant and outcome. The observed benefits of co-transplanting OECs with fibroblasts in rodent studies has led to human clinical trials omitting purification procedures, resulting in modest functional improvement using both OM and OB cell preparations either on their own or in combinatorial treatment respectively, as described above (Tabakow et al., 2013, 2014). Despite the potential for complexity of cell cultures the precise cell types have been rarely well characterized in clinical studies of both OB and OM transplants. A number of different cell types exist alongside OECs *in situ*, in mixed cell transplants it might be possible that cells other than OECs contribute to CNS repair, particularly when derived from the mucosa (Yao et al., 2018). Schwann cells might contaminate OEC transplants as branches of the trigeminal nerve are present in the OM and OB. Defining Schwann cell contribution is difficult because Schwann cells and OECs both express the same markers *in vitro* (Chen C. R. et al., 2014, Chen L. et al., 2014). Additionally, using different cell culture protocols to OECs, MSCs cultured from the lamina propria of human OM have been shown to promote CNS remyelination both *in vitro* and *in vivo* in rat models of SCI (Lindsay et al., 2013, 2017). Conversely, it is also possible that without purification undesirable cells might remain in the cell transplant. Dlouhy et al. (2014) reported a patient with SCI developed a spinal cord tumor mass 8 years after intraspinal transplantation of autologous undissociated OM tissue. It is worth noting that this transplant was neither characterized nor cultured prior to transplantation and other clinical studies using dissociated OM cell preparations have been shown to be relatively safe (Feron et al., 2005; Mackay-Sim et al., 2008; Tabakow et al., 2013).

Nevertheless, regulatory requirements for clinical application of OEC transplants in SCI will require a high level of therapeutic specification and reproducibility. The future development of human OEC transplants will therefore require identification of the cell types and proportions in the transplant that leads to repair. If we are to use OEC transplants for human transplantation, we need to understand what constitutes the human cell cultures and the importance of additional cell types in the cultures for SCI repair.

### Cell Dosage in Transplants

**FIGURE 5:**
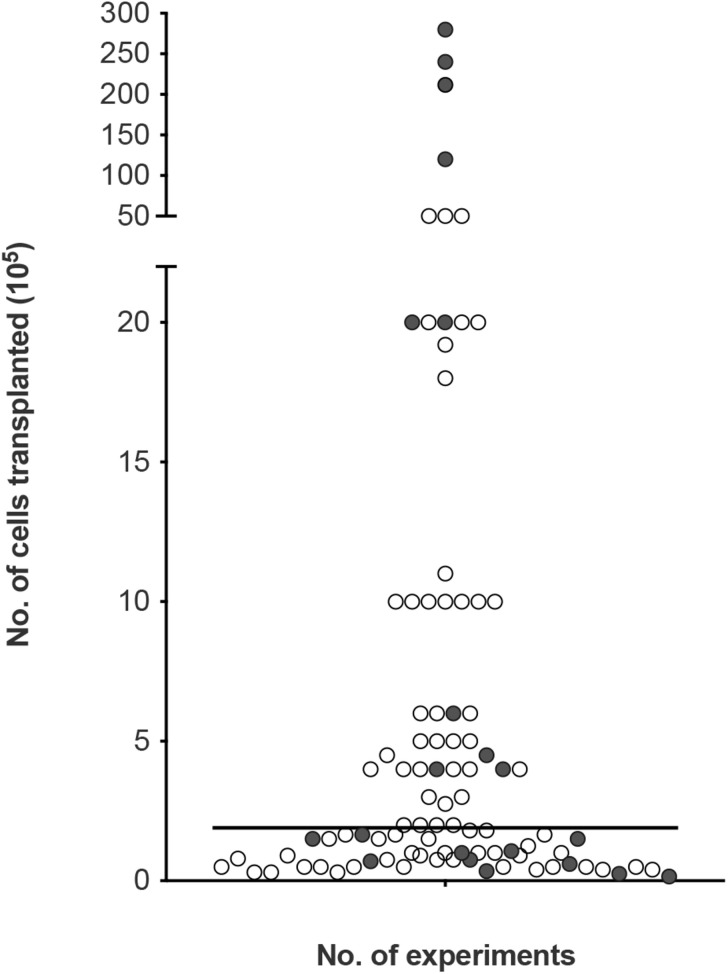
Assessment of cellular dose of olfactory cell transplants in spinal cord injury. A total of 94 experiments were included in the analysis (4 experiments did not provide details of the about the number of cells transplanted). The bar represents the median value of the data analyzed (1.8 × 10^5^ or 180,000 cells in total in a transplant, irrespective of cell mixture proportions). The empty circles indicate the positive outcome experiments and the filled circles indicate the negative outcome experiments.

To look at the potential effect of the cell dose in olfactory cell transplants rather than the proportions, we reviewed the number of experiments reporting the therapeutic outcome compared to the absolute number of transplanted cells (i.e., the total number of cells, not limited to OEC component of the cell cultures, Figure 5). The number of cells transplanted ranged from 15,000 to 28 million cells across the different species. The trend in the data collected shows the number of cells required for therapeutic repair might be low as the majority of the studies reviewed, transplanted a total of 500,000 cells or less *n* = 67 (71.3%), of which 79.1% (*n* = 53) reported neurological improvement. In comparison, transplantation of greater than 500,000 cells resulted in slightly fewer studies reporting neurological improvement, 74.1% (*n* = 20) of studies. We found the median dose of cells transplanted was ∼200,000 cells.

Very few studies looked at the effect of cell dose on neurological improvement. Although, ∼200,000 cells might be sufficient for transplant-mediated repair it might not be the optimal dose of cells. A recent study included in the analysis of this review, compared different dosages of OM cell preparations in a contusion injury model in rats. Treatment groups included 200,000, 500,000, 1 million and >1 million cells. While, all transplanted groups led to higher functional recovery compared to the control injury-only group, 500,000 transplanted cells showed the best improvement in hind-limb motor recovery among the treated groups (Muniswami and Tharion, 2018). This study suggests the efficacy of olfactory transplants may be dose dependent but more studies comparing the dose of cells in the same experimental paradigm are required to determine the optimum cell dose. It is possible the cell dose might be contingent upon the size of the species and the size of the lesion. Furthermore, there was no clear relationship between studies reporting a lack of therapeutic benefit with the total number of cells transplanted. This may be due to a myriad of confounding factors, the most obvious being the great variability in cell culture techniques between different laboratories.

Effective cell doses for human SCI have not been established. Since the majority of our data collected relates to studies in rats, the effective dose in human cell transplantation will likely be higher. In clinical studies neurological improvements were observed when the number of cells transplanted ranged from 500,000 (Tabakow et al., 2014) to ∼2 million cells (Tabakow et al., 2013). Increasing the number of cells did not appear to be linked with more positive results. When three patients with thoracic paraplegia received 1.8, 1.9, and 21.2 million cells, functional improvements were only observed in the two patients who received the lower cell doses. The third patient receiving the higher cells dose in comparison showed minimal functional improvement (Tabakow et al., 2013). In another clinical study, 12, 24, and 28 million cells were transplanted in three paraplegic patients. No significant functional recovery was observed 3 years after transplantation except in the patient who received the lower cell dose showed some improvement in light touch and pin prick sensitivity (Feron et al., 2005; Mackay-Sim et al., 2008). It may be the case that the capacity for OEC transplant-mediated repair is reduced/minimal using higher cell numbers in human. This is consistent with a meta-analysis conducted by Watzlawick et al. (2016) who demonstrated that cell numbers above the optimum dose of 180,000 cells were associated with reduced locomotor outcome in rat models of traumatic SCI. The declining benefit-risk ratio linked to higher cell doses was suggested to be due to the increasing neurotoxic effects exerted by the requirement for higher volumes of injections. Higher cell doses are likely to require larger injection volumes and the delivery of such volumes have the potential to cause further neurological injury to the already injured spinal cord due to the build-up of fluid pressure (Guest et al., 2011). Nonetheless, with so few studies transplanting high cell numbers it would be difficult to claim that higher cell numbers in the transplant are associated with lack of neurological improvement in humans but if a ceiling to the beneficial effect exists it requires further investigation. Additionally, a recent review (Reshamwala et al., 2019) suggested the survival of OECs after transplantation was likely influenced by the number/concentration of transplanted cells. It was suggested that survival was highest when using transplants containing low cell densities. Higher cell densities were linked to negative outcomes possibly due to the increased shear stress and physical damage to the cells during injection procedure. Approaches to improving cell survival will likely lead to improvements in functional outcomes.

### Limitations of the Review

The main limitation of the present study is the great heterogeneity in methodology (cell culture techniques, animal injury models, etc.) and outcome measures (axon regeneration, myelination, and motor recovery, etc.) across studies, making accurate comparisons challenging. In order to compare results from such a variety of sources the different outcome measures were combined into a general classification of positive and negative outcomes. It is possible that this binary classification may mask treatment related effects on specific outcomes but without the standardization of methodology and outcome measures accurate interpretation will always be difficult. Additionally, the design of our study meant that statistical analysis was not possible, the trends in the data was measured instead. In summary, the review provides a broad understanding of the characterization of OEC transplants used for the treatment of SCI and highlights some of the outstanding questions that need to be resolved to maximize treatments for human SCI.

## Conclusion and Recommendations

Over the years the olfactory system has been proposed as a useful tissue to harvest cells for transplant-mediated repair of SCI. In this review we summarized the current literature relating to the composition, purity and cell dose of OEC cultures used for transplant-mediated repair of SCI. Overall, we found that OEC transplants are not very well characterized and crucial questions about the therapeutic cellular composition, purity and dose remain unanswered. Unless olfactory cell transplants are composed of 100% OECs, additional cells will remain in the transplants and it is presently unknown what effect variations in the cell types and proportions will have *in vivo* and if they contribute to therapeutic outcome. The future development of human OEC transplants for clinical application in SCI repair will require characterization and identification of the regenerative components of OEC transplants as well as the purity and cell dose. These are important factors that must be optimized in the clinical translation of any cellular strategy. A better understanding of the components of OEC transplants that leads to repair will further strengthen the effectiveness of cell transplants for the treatment of SCI and improve the clinical outcomes.

A wealth of preclinical data generated using rodent studies has been valuable in demonstrating the benefits of olfactory tissue culture transplantation. However, this review highlights the lack of studies involving human OECs. Human OECs should be studied in their own right, and for ethical reasons, OM is the more likely candidate for initial transplantation in patients. Future studies should therefore endeavor to characterize and identify the regenerative cell types in the OM and look to find optimal cell culture mixes in more clinically relevant injury models such as spinal cord contusion and compression. These cell types should be tested *in vitro*, such as in neurite outgrowth or organotypic slice culture models, and *in vivo*, to identify the active components and estimate the minimum number of cells required for clinical effectiveness. The active cell types should then be cultured and expanded under Good Manufacturing Practice guidelines to allow future clinical application.

## Author Contributions

DC provided the idea for the review. MM searched the literature, prepared the figures, and structured and wrote the manuscript. DC and PF contributed to editing and approval of the final manuscript. All authors contributed to the article and approved the submitted version.

## Conflict of Interest

The authors declare that the research was conducted in the absence of any commercial or financial relationships that could be construed as a potential conflict of interest.

## Publisher’s Note

All claims expressed in this article are solely those of the authors and do not necessarily represent those of their affiliated organizations, or those of the publisher, the editors and the reviewers. Any product that may be evaluated in this article, or claim that may be made by its manufacturer, is not guaranteed or endorsed by the publisher.

## References

[B1] Akiyama Y. Lankford K. Radtke C. Greer C. A. Kocsis J. D. (2004). Remyelination of spinal cord axons by olfactory ensheathing cells and Schwann cells derived from a transgenic rat expressing alkaline phosphatase marker gene. *Neuron Glia Biol.* 1 47–55. 10.1017/S1740925X04000079 16799702 PMC1482729

[B2] Amemori T. Jendelova P. Ruzickova K. Arboleda D. Sykova E. (2010). Co-transplantation of olfactory ensheathing glia and mesenchymal stromal cells does not have synergistic effects after spinal cord injury in the rat. *Cytotherapy* 12 212–225. 10.3109/14653240903440103 20196694

[B3] Andrews M. R. Stelzner D. J. (2004). Modification of the regenerative response of dorsal column axons by olfactory ensheathing cells or peripheral axotomy in adult rat. *Exp. Neurol.* 190 311–327. 10.1016/j.expneurol.2004.08.011 15530871

[B4] Andrews M. R. Stelzner D. J. (2007). Evaluation of olfactory ensheathing and Schwann cells after implantation into a dorsal injury of adult rat spinal cord. *J. Neurotrauma* 24 1773–1792. 10.1089/neu.2007.0353 18001205

[B5] Andrews P. J. Poirrier A. L. Lund V. J. Choi D. (2016). Safety of human olfactory mucosal biopsy for the purpose of olfactory ensheathing cell harvest and nerve repair: a prospective controlled study in patients undergoing endoscopic sinus surgery. *Rhinology* 54 183–191. 10.4193/Rhino15.365 27107010

[B6] Assinck P. Duncan G. J. Hilton B. J. Plemel J. R. Tetzlaff W. (2017). Cell transplantation therapy for spinal cord injury. *Nat. Neurosci.* 20 637–647. 28440805 10.1038/nn.4541

[B7] Barakat D. J. Gaglani S. M. Neravetla S. R. Sanchez A. R. Andrade C. M. Pressman Y. (2005). Survival, integration, and axon growth support of glia transplanted into the chronically contused spinal cord. *Cell Transplant.* 14 225–240. 10.3727/000000005783983106 15929557

[B8] Barnett S. C. Alexander C. L. Iwashita Y. Gilson J. M. Crowther J. Clark L. (2000). Identification of a human olfactory ensheathing cell that can effect transplant-mediated remyelination of demyelinated CNS axons. *Brain* 123(Pt 8) 1581–1588. 10.1093/brain/123.8.1581 10908188

[B9] Borgmann-Winter K. Willard S. L. Sinclair D. Mirza N. Turetsky B. Berretta S. (2015). Translational potential of olfactory mucosa for the study of neuropsychiatric illness. *Transl. Psychiatry* 5:e527. 10.1038/tp.2014.141 25781226 PMC4354342

[B10] Bretzner F. Liu J. Currie E. Roskams A. J. Tetzlaff W. (2008). Undesired effects of a combinatorial treatment for spinal cord injury–transplantation of olfactory ensheathing cells and BDNF infusion to the red nucleus. *Eur. J. Neurosci.* 28 1795–1807. 10.1111/j.1460-9568.2008.06462.x 18973595

[B11] Bretzner F. Plemel J. R. Liu J. Richter M. Roskams A. J. Tetzlaff W. (2010). Combination of olfactory ensheathing cells with local versus systemic cAMP treatment after a cervical rubrospinal tract injury. *J. Neurosci. Res.* 88 2833–2846. 10.1002/jnr.22440 20568293

[B12] Cao L. Liu L. Chen Z. Y. Wang L. M. Ye J. L. Qiu H. Y. (2004). Olfactory ensheathing cells genetically modified to secrete GDNF to promote spinal cord repair. *Brain* 127 535–549. 10.1093/brain/awh072 14691064

[B13] Cao L. Su Z. Zhou Q. Lv B. Liu X. Jiao L. (2006). Glial cell line-derived neurotrophic factor promotes olfactory ensheathing cells migration. *Glia* 54 536–544. 16906542 10.1002/glia.20403

[B14] Carwardine D. Prager J. Neeves J. Muir E. M. Uney J. Granger N. (2017). Transplantation of canine olfactory ensheathing cells producing chondroitinase ABC promotes chondroitin sulphate proteoglycan digestion and axonal sprouting following spinal cord injury. *PLoS One* 12:e0188967. 10.1371/journal.pone.0188967 29228020 PMC5724818

[B15] Chen C. R. Kachramanoglou C. Li D. Andrews P. Choi D. (2014). Anatomy and cellular constituents of the human olfactory mucosa: a review. *J. Neurol. Surg. B Skull Base* 75 293–300. 10.1055/s-0033-1361837 25302141 PMC4176544

[B16] Chen L. Huang H. Xi H. Zhang F. Liu Y. Chen D. (2014). A prospective randomized double-blind clinical trial using a combination of olfactory ensheathing cells and Schwann cells for the treatment of chronic complete spinal cord injuries. *Cell Transplant.* 23(Suppl. 1) S35–S44. 10.3727/096368914X685014 25333925

[B17] Choi D. Law S. Raisman G. Li D. (2008a). Olfactory ensheathing cells in the nasal mucosa of the rat and human. *Br. J. Neurosurg.* 22 301–302. 10.1080/02688690701883442 18348034

[B18] Choi D. Li D. Law S. Powell M. Raisman G. (2008b). A prospective observational study of the yield of olfactory ensheathing cells cultured from biopsies of septal nasal mucosa. *Neurosurgery* 62 1140–1145. 10.1227/01.neu.0000325876.90623.df 18580812

[B19] Chuah M. I. Hale D. M. West A. K. (2011). Interaction of olfactory ensheathing cells with other cell types in vitro and after transplantation: glial scars and inflammation. *Exp. Neurol.* 229 46–53. 10.1016/j.expneurol.2010.08.012 20713050

[B20] Chung R. S. Woodhouse A. Fung S. Dickson T. C. West A. K. Vickers J. C. (2004). Olfactory ensheathing cells promote neurite sprouting of injured axons in vitro by direct cellular contact and secretion of soluble factors. *Cell. Mol. Life Sci.* 61 1238–1245. 10.1007/s00018-004-4026-y 15141309 PMC11146019

[B21] Collins A. Li D. Liadi M. Tabakow P. Fortuna W. Raisman G. (2018). Partial recovery of proprioception in rats with dorsal root injury after human olfactory bulb cell transplantation. *J. Neurotrauma* 35 1367–1378. 10.1089/neu.2017.5273 29285976

[B22] Coutts D. J. Humphries C. E. Zhao C. Plant G. W. Franklin R. J. (2013). Embryonic-derived olfactory ensheathing cells remyelinate focal areas of spinal cord demyelination more efficiently than neonatal or adult-derived cells. *Cell Transplant.* 22 1249–1261. 23031825 10.3727/096368912X656153

[B23] Deng C. Gorrie C. Hayward I. Elston B. Venn M. Mackay-Sim A. (2006). Survival and migration of human and rat olfactory ensheathing cells in intact and injured spinal cord. *J. Neurosci. Res.* 83 1201–1212. 10.1002/jnr.20817 16498634

[B24] Deumens R. Koopmans G. C. Honig W. M. Maquet V. Jerome R. Steinbusch H. W. (2006). Chronically injured corticospinal axons do not cross large spinal lesion gaps after a multifactorial transplantation strategy using olfactory ensheathing cell/olfactory nerve fibroblast-biomatrix bridges. *J. Neurosci. Res.* 83 811–820. 16477623 10.1002/jnr.20768

[B25] Dlouhy B. J. Awe O. Rao R. C. Kirby P. A. Hitchon P. W. (2014). Autograft-derived spinal cord mass following olfactory mucosal cell transplantation in a spinal cord injury patient: case report. *J. Neurosurg. Spine* 21 618–622. 10.3171/2014.5.SPINE13992 25002238

[B26] Doucette R. (1990). Glial influences on axonal growth in the primary olfactory system. *Glia* 3 433–449. 10.1002/glia.440030602 2148546

[B27] Doucette R. (1991). PNS-CNS transitional zone of the first cranial nerve. *J. Comp. Neurol.* 312 451–466. 10.1002/cne.903120311 1748741

[B28] Dunning M. D. Lakatos A. Loizou L. Kettunen M. ffrench-Constant C. Brindle K. M. (2004). Superparamagnetic iron oxide-labeled Schwann cells and olfactory ensheathing cells can be traced in vivo by magnetic resonance imaging and retain functional properties after transplantation into the CNS. *J. Neurosci.* 24 9799–9810. 10.1523/JNEUROSCI.3126-04.2004 15525765 PMC6730225

[B29] Ekberg J. A. Amaya D. Mackay-Sim A. St John J. A. (2012). The migration of olfactory ensheathing cells during development and regeneration. *Neurosignals* 20 147–158. 10.1159/000330895 22456085

[B30] Ekberg J. A. St John J. A. (2014). Crucial roles for olfactory ensheathing cells and olfactory mucosal cells in the repair of damaged neural tracts. *Anat. Rec.* 297 121–128. 10.1002/ar.22803 24293406

[B31] Feron F. Perry C. Cochrane J. Licina P. Nowitzke A. Urquhart S. (2005). Autologous olfactory ensheathing cell transplantation in human spinal cord injury. *Brain* 128 2951–2960. 10.1093/brain/awh657 16219671

[B32] Field P. Li Y. Raisman G. (2003). Ensheathment of the olfactory nerves in the adult rat. *J. Neurocytol.* 32 317–324. 10.1023/b:neur.0000010089.37032.48 14724393

[B33] Gomez V. M. Averill S. King V. Yang Q. Doncel Perez E. Chacon S. C. (2003). Transplantation of olfactory ensheathing cells fails to promote significant axonal regeneration from dorsal roots into the rat cervical cord. *J. Neurocytol.* 32 53–70. 10.1023/a:1027328331832 14618101

[B34] Granger N. Blamires H. Franklin R. J. Jeffery N. D. (2012). Autologous olfactory mucosal cell transplants in clinical spinal cord injury: a randomized double-blinded trial in a canine translational model. *Brain* 135 3227–3237. 10.1093/brain/aws268 23169917 PMC3501977

[B35] Graziadei G. A. Graziadei P. P. (1979a). Neurogenesis and neuron regeneration in the olfactory system of mammals. II. Degeneration and reconstitution of the olfactory sensory neurons after axotomy. *J. Neurocytol.* 8 197–213. 10.1007/BF01175561 469573

[B36] Graziadei P. P. Graziadei G. A. (1979b). Neurogenesis and neuron regeneration in the olfactory system of mammals. I. Morphological aspects of differentiation and structural organization of the olfactory sensory neurons. *J. Neurocytol.* 8 1–18. 10.1007/BF01206454 438867

[B37] Graziadei P. P. Okano M. (1979c). Neuronal degeneration and regeneration in the olfactory epithelium of pigeon following transection of the first cranial nerve. *Acta Anat.* 104 220–236. 10.1159/000145070 442974

[B38] Graziadei P. P. Karlan M. S. Graziadei G. A. Bernstein J. J. (1980). Neurogenesis of sensory neurons in the primate olfactory system after section of the fila olfactoria. *Brain Res.* 186 289–300. 10.1016/0006-8993(80)90976-2 6766784

[B39] Gu M. Gao Z. Li X. Zhao F. Guo L. Liu J. (2017). Feasibility of diffusion tensor imaging for assessing functional recovery in rats with olfactory ensheathing cell transplantation after contusive spinal cord injury (SCI). *Med. Sci. Monit.* 23 2961–2971. 10.12659/msm.902126 28623671 PMC5484594

[B40] Guest J. Benavides F. Padgett K. Mendez E. Tovar D. (2011). Technical aspects of spinal cord injections for cell transplantation. Clinical and translational considerations. *Brain Res. Bull.* 84 267–279. 10.1016/j.brainresbull.2010.11.007 21087657

[B41] Guest J. Dietrich W. D. (2015). Commentary regarding the recent publication by Tabakow et al., “Functional regeneration of supraspinal connections in a patient with transected spinal cord following transplantation of bulbar olfactory ensheathing cells with peripheral nerve bridging”. *J. Neurotrauma* 32 1176–1178. 10.3727/096368914X685131 25412291

[B42] Guest J. D. Herrera L. Margitich I. Oliveria M. Marcillo A. Casas C. E. (2008). Xenografts of expanded primate olfactory ensheathing glia support transient behavioral recovery that is independent of serotonergic or corticospinal axonal regeneration in nude rats following spinal cord transection. *Exp. Neurol.* 212 261–274. 10.1016/j.expneurol.2008.03.010 18511045

[B43] Hahn C. G. Han L. Y. Rawson N. E. Mirza N. Borgmann-Winter K. Lenox R. H. (2005). In vivo and in vitro neurogenesis in human olfactory epithelium. *J. Comp. Neurol.* 483 154–163. 10.1002/cne.20424 15678478

[B44] Holbrook E. H. Rebeiz L. Schwob J. E. (2016). Office-based olfactory mucosa biopsies. *Int. Forum Allergy Rhinol.* 6 646–653. 10.1002/alr.21711 26833660 PMC4921276

[B45] Holbrook E. H. Wu E. Curry W. T. Lin D. T. Schwob J. E. (2011). Immunohistochemical characterization of human olfactory tissue. *Laryngoscope* 121 1687–1701. 10.1002/lary.21856 21792956 PMC3181071

[B46] Ibrahim A. Li D. Collins A. Tabakow P. Raisman G. Li Y. (2014). Comparison of olfactory bulbar and mucosal cultures in a rat rhizotomy model. *Cell Transplant.* 23 1465–1470. 10.3727/096368913X676213 24380436

[B47] Ibrahim A. G. Kirkwood P. A. Raisman G. Li Y. (2009). Restoration of hand function in a rat model of repair of brachial plexus injury. *Brain* 132 1268–1276. 10.1093/brain/awp030 19286693

[B48] Jani H. R. Raisman G. (2004). Ensheathing cell cultures from the olfactory bulb and mucosa. *Glia* 47 130–137. 10.1002/glia.20038 15185392

[B49] Kalincik T. Jozefcikova K. Sutharsan R. Mackay-Sim A. Carrive P. Waite P. M. (2010b). Selected changes in spinal cord morphology after T4 transection and olfactory ensheathing cell transplantation. *Auton. Neurosci.* 158 31–38. 10.1016/j.autneu.2010.05.011 20594923

[B50] Kalincik T. Choi E. A. Feron F. Bianco J. Sutharsan R. Hayward I. (2010a). Olfactory ensheathing cells reduce duration of autonomic dysreflexia in rats with high spinal cord injury. *Auton. Neurosci.* 154 20–29. 19896908 10.1016/j.autneu.2009.10.001

[B51] Kang X. W. Hu J. L. Wang S. K. Wang J. (2015). Effectiveness of muscle basal lamina carrying neural stem cells and olfactory ensheathing cells in spinal cord repair. *Genet. Mol. Res.* 14 13437–13455. 10.4238/2015.October.28.5 26535658

[B52] Kawaja M. D. Boyd J. G. Smithson L. J. Jahed A. Doucette R. (2009). Technical strategies to isolate olfactory ensheathing cells for intraspinal implantation. *J. Neurotrauma* 26 155–177. 10.1089/neu.2008.0709 19196079

[B53] Keyvan-Fouladi N. Raisman G. Li Y. (2003). Functional repair of the corticospinal tract by delayed transplantation of olfactory ensheathing cells in adult rats. *J. Neurosci.* 23 9428–9434. 10.1523/jneurosci.23-28-09428.2003 14561871 PMC6740581

[B54] Khankan R. R. Griffis K. G. Haggerty-Skeans J. R. Zhong H. Roy R. R. Edgerton V. R. (2016). Olfactory ensheathing cell transplantation after a complete spinal cord transection mediates neuroprotective and immunomodulatory mechanisms to facilitate regeneration. *J. Neurosci.* 36 6269–6286. 10.1523/JNEUROSCI.0085-16.2016 27277804 PMC4899528

[B55] Kondo K. Suzukawa K. Sakamoto T. Watanabe K. Kanaya K. Ushio M. (2010). Age-related changes in cell dynamics of the postnatal mouse olfactory neuroepithelium: cell proliferation, neuronal differentiation, and cell death. *J. Comp. Neurol.* 518 1962–1975. 10.1002/cne.22316 20394053

[B56] Kornack D. R. Rakic P. (2001). The generation, migration, and differentiation of olfactory neurons in the adult primate brain. *Proc. Natl. Acad. Sci. U.S.A.* 98 4752–4757. 10.1073/pnas.081074998 11296302 PMC31906

[B57] Kueh J. L. Raisman G. Li Y. Stevens R. Li D. (2011). Comparison of bulbar and mucosal olfactory ensheathing cells using FACS and simultaneous antigenic bivariate cell cycle analysis. *Glia* 59 1658–1671. 10.1002/glia.21213 21748806

[B58] Lakatos A. Smith P. M. Barnett S. C. Franklin R. J. (2003). Meningeal cells enhance limited CNS remyelination by transplanted olfactory ensheathing cells. *Brain* 126 598–609. 10.1093/brain/awg055 12566281

[B59] Lang B. C. Zhang Z. Lv L. Y. Liu J. Wang T. Y. Yang L. H. (2013). OECs transplantation results in neuropathic pain associated with BDNF regulating ERK activity in rats following cord hemisection. *BMC Neurosci.* 14:80. 10.1186/1471-2202-14-80 23914898 PMC3734147

[B60] Lankford K. L. Sasaki M. Radtke C. Kocsis J. D. (2008). Olfactory ensheathing cells exhibit unique migratory, phagocytic, and myelinating properties in the X-irradiated spinal cord not shared by Schwann cells. *Glia* 56 1664–1678. 10.1002/glia.20718 18551623

[B61] Lee I. H. Bulte J. W. Schweinhardt P. Douglas T. Trifunovski A. Hofstetter C. (2004). In vivo magnetic resonance tracking of olfactory ensheathing glia grafted into the rat spinal cord. *Exp. Neurol.* 187 509–516. 10.1016/j.expneurol.2004.02.007 15144877

[B62] Leung C. T. Coulombe P. A. Reed R. R. (2007). Contribution of olfactory neural stem cells to tissue maintenance and regeneration. *Nat. Neurosci.* 10 720–726. 17468753 10.1038/nn1882

[B63] Leung J. Y. Chapman J. A. Harris J. A. Hale D. Chung R. S. West A. K. (2008). Olfactory ensheathing cells are attracted to, and can endocytose, bacteria. *Cell. Mol. Life Sci.* 65 2732–2739. 10.1007/s00018-008-8184-1 18604629 PMC11131851

[B64] Li B. C. Li Y. Chen L. F. Chang J. Y. Duan Z. X. (2011). Olfactory ensheathing cells can reduce the tissue loss but not the cavity formation in contused spinal cord of rats. *J. Neurol. Sci.* 303 67–74. 10.1016/j.jns.2011.01.013 21306739

[B65] Li B. C. Xu C. Zhang J. Y. Li Y. Duan Z. X. (2012). Differing Schwann cells and olfactory ensheathing cells behaviors, from interacting with astrocyte, produce similar improvements in contused rat spinal cord’s motor function. *J. Mol. Neurosci.* 48 35–44. 10.1007/s12031-012-9740-6 22407596

[B66] Li Y. Carlstedt T. Berthold C. H. Raisman G. (2004). Interaction of transplanted olfactory-ensheathing cells and host astrocytic processes provides a bridge for axons to regenerate across the dorsal root entry zone. *Exp. Neurol.* 188 300–308. 10.1016/j.expneurol.2004.04.021 15246830

[B67] Li Y. Decherchi P. Raisman G. (2003). Transplantation of olfactory ensheathing cells into spinal cord lesions restores breathing and climbing. *J. Neurosci.* 23 727–731. 10.1523/JNEUROSCI.23-03-00727.2003 12574399 PMC6741902

[B68] Li Y. Field P. M. Raisman G. (1997). Repair of adult rat corticospinal tract by transplants of olfactory ensheathing cells. *Science* 277 2000–2002. 9302296 10.1126/science.277.5334.2000

[B69] Li Y. Field P. M. Raisman G. (1998). Regeneration of adult rat corticospinal axons induced by transplanted olfactory ensheathing cells. *J. Neurosci.* 18 10514–10524. 10.1523/jneurosci.18-24-10514.1998 9852589 PMC6793366

[B70] Li Y. Li D. Raisman G. (2016). Functional repair of rat corticospinal tract lesions does not require permanent survival of an immunoincompatible transplant. *Cell Transplant.* 25 293–299. 10.3727/096368915x688551 26132822

[B71] Lindsay S. L. Johnstone S. A. Mountford J. C. Sheikh S. Allan D. B. Clark L. (2013). Human mesenchymal stem cells isolated from olfactory biopsies but not bone enhance CNS myelination in vitro. *Glia* 61 368–382. 23281012 10.1002/glia.22440

[B72] Lindsay S. L. Riddell J. S. Barnett S. C. (2010). Olfactory mucosa for transplant-mediated repair: a complex tissue for a complex injury? *Glia* 58 125–134. 10.1002/glia.20917 19606497

[B73] Lindsay S. L. Toft A. Griffin J. Emraja A. M. M. Barnett S. C. Riddell J. S. (2017). Human olfactory mesenchymal stromal cell transplants promote remyelination and earlier improvement in gait co-ordination after spinal cord injury. *Glia* 65 639–656. 10.1002/glia.23117 28144983 PMC5324664

[B74] Lipson A. C. Widenfalk J. Lindqvist E. Ebendal T. Olson L. (2003). Neurotrophic properties of olfactory ensheathing glia. *Exp. Neurol.* 180 167–171. 12684030 10.1016/s0014-4886(02)00058-4

[B75] Lopez-Vales R. Fores J. Verdu E. Navarro X. (2006). Acute and delayed transplantation of olfactory ensheathing cells promote partial recovery after complete transection of the spinal cord. *Neurobiol. Dis.* 21 57–68. 10.1016/j.nbd.2005.06.011 16051494

[B76] Lopez-Vales R. Garcia-Alias G. Fores J. Navarro X. Verdu E. (2004). Increased expression of cyclo-oxygenase 2 and vascular endothelial growth factor in lesioned spinal cord by transplanted olfactory ensheathing cells. *J. Neurotrauma* 21 1031–1043. 10.1089/0897715041651105 15319002

[B77] Lu J. Feron F. Ho S. M. Mackay-Sim A. Waite P. M. (2001). Transplantation of nasal olfactory tissue promotes partial recovery in paraplegic adult rats. *Brain Res.* 889 344–357. 10.1016/s0006-8993(00)03235-2 11166728

[B78] Lu J. Feron F. Mackay-Sim A. Waite P. M. (2002). Olfactory ensheathing cells promote locomotor recovery after delayed transplantation into transected spinal cord. *Brain* 125 14–21. 10.1093/brain/awf014 11834589

[B79] Lu P. Yang H. Culbertson M. Graham L. Roskams A. J. Tuszynski M. H. (2006). Olfactory ensheathing cells do not exhibit unique migratory or axonal growth-promoting properties after spinal cord injury. *J. Neurosci.* 26 11120–11130. 10.1523/jneurosci.3264-06.2006 17065452 PMC6674649

[B80] Mackay-Sim A. Feron F. Cochrane J. Bassingthwaighte L. Bayliss C. Davies W. (2008). Autologous olfactory ensheathing cell transplantation in human paraplegia: a 3-year clinical trial. *Brain* 131 2376–2386. 10.1093/brain/awn173 18689435 PMC2525447

[B81] Mackay-Sim A. Kittel P. W. (1991). On the life span of olfactory receptor neurons. *Eur. J. Neurosci.* 3 209–215. 12106197 10.1111/j.1460-9568.1991.tb00081.x

[B82] Mayeur A. Duclos C. Honore A. Gauberti M. Drouot L. do Rego J. C. (2013). Potential of olfactory ensheathing cells from different sources for spinal cord repair. *PLoS One* 8:e62860. 10.1371/journal.pone.0062860 23638158 PMC3634744

[B83] Mobley A. S. Rodriguez-Gil D. J. Imamura F. Greer C. A. (2014). Aging in the olfactory system. *Trends Neurosci.* 37 77–84. 24361044 10.1016/j.tins.2013.11.004 PMC3947771

[B84] Muniswami D. M. Tharion G. (2018). Functional recovery following the transplantation of olfactory ensheathing cells in rat spinal cord injury model. *Asian Spine J.* 12 998–1009. 10.31616/asj.2018.12.6.998 30322257 PMC6284116

[B85] Nakhjavan-Shahraki B. Yousefifard M. Rahimi-Movaghar V. Baikpour M. Nasirinezhad F. Safari S. (2018). Transplantation of olfactory ensheathing cells on functional recovery and neuropathic pain after spinal cord injury; systematic review and meta-analysis. *Sci. Rep.* 8:325. 10.1038/s41598-017-18754-4 29321494 PMC5762885

[B86] Nash H. H. Borke R. C. Anders J. J. (2002). Ensheathing cells and methylprednisolone promote axonal regeneration and functional recovery in the lesioned adult rat spinal cord. *J. Neurosci.* 22 7111–7120. 10.1523/JNEUROSCI.22-16-07111.2002 12177207 PMC6757894

[B87] Nazareth L. Lineburg K. E. Chuah M. I. Tello Velasquez J. Chehrehasa F. St John J. A. (2015). Olfactory ensheathing cells are the main phagocytic cells that remove axon debris during early development of the olfactory system. *J. Comp. Neurol.* 523 479–494. 10.1002/cne.23694 25312022

[B88] Novikova L. N. Lobov S. Wiberg M. Novikov L. N. (2011). Efficacy of olfactory ensheathing cells to support regeneration after spinal cord injury is influenced by method of culture preparation. *Exp. Neurol.* 229 132–142. 10.1016/j.expneurol.2010.09.021 20932826

[B89] Panni P. Ferguson I. A. Beacham I. Mackay-Sim A. Ekberg J. A. K. St John J. A. (2013). Phagocytosis of bacteria by olfactory ensheathing cells and Schwann cells. *Neurosci. Lett.* 539 65–70. 10.1016/j.neulet.2013.01.052 23415759

[B90] Pearse D. D. Marcillo A. E. Oudega M. Lynch M. P. Wood P. M. Bunge M. B. (2004). Transplantation of Schwann cells and olfactory ensheathing glia after spinal cord injury: does pretreatment with methylprednisolone and interleukin-10 enhance recovery? *J. Neurotrauma* 21 1223–1239. 10.1089/neu.2004.21.1223 15453992

[B91] Pearse D. D. Sanchez A. R. Pereira F. C. Andrade C. M. Puzis R. Pressman Y. (2007). Transplantation of Schwann cells and/or olfactory ensheathing glia into the contused spinal cord: survival, migration, axon association, and functional recovery. *Glia* 55 976–1000. 17526000 10.1002/glia.20490

[B92] Plant G. W. Christensen C. L. Oudega M. Bunge M. B. (2003). Delayed transplantation of olfactory ensheathing glia promotes sparing/regeneration of supraspinal axons in the contused adult rat spinal cord. *J. Neurotrauma* 20 1–16. 10.1089/08977150360517146 12614584

[B93] Radtke C. Akiyama Y. Brokaw J. Lankford K. L. Wewetzer K. Fodor W. L. (2004). Remyelination of the nonhuman primate spinal cord by transplantation of H-transferase transgenic adult pig olfactory ensheathing cells. *FASEB J.* 18 335–337. 10.1096/fj.03-0214fje 14657003 PMC2605365

[B94] Raisman G. Li Y. (2007). Repair of neural pathways by olfactory ensheathing cells. *Nat. Rev. Neurosci.* 8 312–319. 17342173 10.1038/nrn2099

[B95] Ramer L. M. Au E. Richter M. W. Liu J. Tetzlaff W. Roskams A. J. (2004). Peripheral olfactory ensheathing cells reduce scar and cavity formation and promote regeneration after spinal cord injury. *J. Comp. Neurol.* 473 1–15. 10.1002/cne.20049 15067714

[B96] Ramon-Cueto A. Cordero M. I. Santos-Benito F. F. Avila J. (2000). Functional recovery of paraplegic rats and motor axon regeneration in their spinal cords by olfactory ensheathing glia. *Neuron* 25 425–435. 10.1016/s0896-6273(00)80905-8 10719896

[B97] Ramon-Cueto A. Nieto-Sampedro M. (1994). Regeneration into the spinal cord of transected dorsal root axons is promoted by ensheathing glia transplants. *Exp. Neurol.* 127 232–244. 10.1006/exnr.1994.1099 8033963

[B98] Ramon-Cueto A. Plant G. W. Avila J. Bunge M. B. (1998). Long-distance axonal regeneration in the transected adult rat spinal cord is promoted by olfactory ensheathing glia transplants. *J. Neurosci.* 18 3803–3815. 9570810 10.1523/JNEUROSCI.18-10-03803.1998 PMC6793168

[B99] Reshamwala R. Shah M. Belt L. Ekberg J. A. K. St John J. A. (2020). Reliable cell purification and determination of cell purity: crucial aspects of olfactory ensheathing cell transplantation for spinal cord repair. *Neural Regen. Res.* 15 2016–2026. 10.4103/1673-5374.282218 32394949 PMC7716040

[B100] Reshamwala R. Shah M. St John J. Ekberg J. (2019). Survival and integration of transplanted olfactory ensheathing cells are crucial for spinal cord injury repair: insights from the last 10 years of animal model studies. *Cell Transplant.* 28 132S–159S. 10.1177/0963689719883823 31726863 PMC7016467

[B101] Riddell J. S. Enriquez-Denton M. Toft A. Fairless R. Barnett S. C. (2004). Olfactory ensheathing cell grafts have minimal influence on regeneration at the dorsal root entry zone following rhizotomy. *Glia* 47 150–167. 10.1002/glia.20041 15185394

[B102] Roet K. C. Verhaagen J. (2014). Understanding the neural repair-promoting properties of olfactory ensheathing cells. *Exp. Neurol.* 261C 594–609. 10.1016/j.expneurol.2014.05.007 24842489

[B103] Sasaki M. Black J. A. Lankford K. L. Tokuno H. A. Waxman S. G. Kocsis J. D. (2006). Molecular reconstruction of nodes of Ranvier after remyelination by transplanted olfactory ensheathing cells in the demyelinated spinal cord. *J. Neurosci.* 26 1803–1812. 10.1523/JNEUROSCI.3611-05.2006 16467529 PMC2605396

[B104] Sasaki M. Lankford K. L. Zemedkun M. Kocsis J. D. (2004). Identified olfactory ensheathing cells transplanted into the transected dorsal funiculus bridge the lesion and form myelin. *J. Neurosci.* 24 8485–8493. 10.1523/JNEUROSCI.1998-04.2004 15456822 PMC2605369

[B105] Schwob J. E. Jang W. Holbrook E. H. Lin B. Herrick D. B. Peterson J. N. (2017). Stem and progenitor cells of the mammalian olfactory epithelium: taking poietic license. *J. Comp. Neurol.* 525 1034–1054. 10.1002/cne.24105 27560601 PMC5805156

[B106] Schwob J. E. Youngentob S. L. Mezza R. C. (1995). Reconstitution of the rat olfactory epithelium after methyl bromide-induced lesion. *J. Comp. Neurol.* 359 15–37. 8557844 10.1002/cne.903590103

[B107] Smith P. M. Lakatos A. Barnett S. C. Jeffery N. D. Franklin R. J. (2002). Cryopreserved cells isolated from the adult canine olfactory bulb are capable of extensive remyelination following transplantation into the adult rat CNS. *Exp. Neurol.* 176 402–406. 10.1006/exnr.2002.7936 12359182

[B108] Smith P. M. Sim F. J. Barnett S. C. Franklin R. J. (2001). SCIP/Oct-6, Krox-20, and desert hedgehog mRNA expression during CNS remyelination by transplanted olfactory ensheathing cells. *Glia* 36 342–353. 10.1002/glia.1121 11746771

[B109] Stamegna J. C. Felix M. S. Roux-Peyronnet J. Rossi V. Feron F. Gauthier P. (2011). Nasal OEC transplantation promotes respiratory recovery in a subchronic rat model of cervical spinal cord contusion. *Exp. Neurol.* 229 120–131. 10.1016/j.expneurol.2010.07.002 20633558

[B110] Steward O. Sharp K. Selvan G. Hadden A. Hofstadter M. Au E. (2006). A re-assessment of the consequences of delayed transplantation of olfactory lamina propria following complete spinal cord transection in rats. *Exp. Neurol.* 198 483–499. 10.1016/j.expneurol.2005.12.034 16494866

[B111] Su Z. Chen J. Qiu Y. Yuan Y. Zhu F. Zhu Y. (2013). Olfactory ensheathing cells: the primary innate immunocytes in the olfactory pathway to engulf apoptotic olfactory nerve debris. *Glia* 61 490–503. 10.1002/glia.22450 23339073

[B112] Tabakow P. Jarmundowicz W. Czapiga B. Fortuna W. Miedzybrodzki R. Czyz M. (2013). Transplantation of autologous olfactory ensheathing cells in complete human spinal cord injury. *Cell Transplant.* 22 1591–1612. 10.3727/096368912x663532 24007776

[B113] Tabakow P. Raisman G. Fortuna W. Czyz M. Huber J. Li D. (2014). Functional regeneration of supraspinal connections in a patient with transected spinal cord following transplantation of bulbar olfactory ensheathing cells with peripheral nerve bridging. *Cell Transplant.* 23 1631–1655. 10.3727/096368914x685131 25338642

[B114] Takami T. Oudega M. Bates M. L. Wood P. M. Kleitman N. Bunge M. B. (2002). Schwann cell but not olfactory ensheathing glia transplants improve hindlimb locomotor performance in the moderately contused adult rat thoracic spinal cord. *J. Neurosci.* 22 6670–6681. 12151546 10.1523/JNEUROSCI.22-15-06670.2002 PMC6758124

[B115] Takeoka A. Jindrich D. L. Munoz-Quiles C. Zhong H. van den Brand R. Pham D. L. (2011). Axon regeneration can facilitate or suppress hindlimb function after olfactory ensheathing glia transplantation. *J. Neurosci.* 31 4298–4310. 10.1523/jneurosci.4967-10.2011 21411671 PMC3327612

[B116] Tennent R. Chuah M. I. (1996). Ultrastructural study of ensheathing cells in early development of olfactory axons. *Brain Res. Dev. Brain Res.* 95 135–139. 8873986 10.1016/0165-3806(96)00091-0

[B117] Tetzlaff W. Okon E. B. Karimi-Abdolrezaee S. Hill C. E. Sparling J. S. Plemel J. R. (2011). A systematic review of cellular transplantation therapies for spinal cord injury. *J. Neurotrauma* 28 1611–1682. 20146557 10.1089/neu.2009.1177 PMC3143488

[B118] Toft A. Scott D. T. Barnett S. C. Riddell J. S. (2007). Electrophysiological evidence that olfactory cell transplants improve function after spinal cord injury. *Brain* 130 970–984. 10.1093/brain/awm040 17438017

[B119] Toft A. Tome M. Barnett S. C. Riddell J. S. (2013). A comparative study of glial and non-neural cell properties for transplant-mediated repair of the injured spinal cord. *Glia* 61 513–528. 10.1002/glia.22452 23322541

[B120] Torres-Espin A. Hernandez J. Navarro X. (2013). Gene expression changes in the injured spinal cord following transplantation of mesenchymal stem cells or olfactory ensheathing cells. *PLoS One* 8:e76141. 10.1371/journal.pone.0076141 24146830 PMC3795752

[B121] Torres-Espin A. Redondo-Castro E. Hernandez J. Navarro X. (2014). Bone marrow mesenchymal stromal cells and olfactory ensheathing cells transplantation after spinal cord injury–a morphological and functional comparison in rats. *Eur. J. Neurosci.* 39 1704–1717. 10.1111/ejn.12542 24635194

[B122] Verdu E. Garcia-Alias G. Fores J. Lopez-Vales R. Navarro X. (2003). Olfactory ensheathing cells transplanted in lesioned spinal cord prevent loss of spinal cord parenchyma and promote functional recovery. *Glia* 42 275–286. 10.1002/glia.10217 12673833

[B123] Wang G. Ao Q. Gong K. Zuo H. Gong Y. Zhang X. (2010). Synergistic effect of neural stem cells and olfactory ensheathing cells on repair of adult rat spinal cord injury. *Cell Transplant.* 19 1325–1337. 10.3727/096368910X505855 20447345

[B124] Watzlawick R. Rind J. Sena E. S. Brommer B. Zhang T. Kopp M. A. (2016). Olfactory ensheathing cell transplantation in experimental spinal cord injury: effect size and reporting bias of 62 experimental treatments: a systematic review and meta-analysis. *PLoS Biol.* 14:e1002468. 10.1371/journal.pbio.1002468 27244556 PMC4886956

[B125] Willison A. G. Smith S. Davies B. M. Kotter M. R. N. Barnett S. C. (2020). A scoping review of trials for cell-based therapies in human spinal cord injury. *Spinal Cord* 58 844–856. 10.1038/s41393-020-0455-1 32249830

[B126] Woodhall E. West A. K. Chuah M. I. (2001). Cultured olfactory ensheathing cells express nerve growth factor, brain-derived neurotrophic factor, glia cell line-derived neurotrophic factor and their receptors. *Brain Res. Mol. Brain Res.* 88 203–213. 11295250 10.1016/s0169-328x(01)00044-4

[B127] Wu A. Lauschke J. L. Gorrie C. A. Cameron N. Hayward I. Mackay-Sim A. (2011). Delayed olfactory ensheathing cell transplants reduce nociception after dorsal root injury. *Exp. Neurol.* 229 143–157. 10.1016/j.expneurol.2010.07.006 20643129

[B128] Yamamoto M. Raisman G. Li D. Li Y. (2009). Transplanted olfactory mucosal cells restore paw reaching function without regeneration of severed corticospinal tract fibres across the lesion. *Brain Res.* 1303 26–31. 10.1016/j.brainres.2009.09.073 19782053

[B129] Yao R. Murtaza M. Velasquez J. T. Todorovic M. Rayfield A. Ekberg J. (2018). Olfactory ensheathing cells for spinal cord injury: sniffing out the issues. *Cell Transplant.* 27 879–889. 10.1177/0963689718779353 29882418 PMC6050914

[B130] Yazdani S. O. Pedram M. Hafizi M. Kabiri M. Soleimani M. Dehghan M. M. (2012). A comparison between neurally induced bone marrow derived mesenchymal stem cells and olfactory ensheathing glial cells to repair spinal cord injuries in rat. *Tissue Cell* 44 205–213. 10.1016/j.tice.2012.03.003 22551686

[B131] Yui S. Ito D. Fujita N. Nishimura R. (2011). Effects of fibroblasts derived from the olfactory bulb and nasal olfactory mucosa on proliferation of olfactory ensheathing cells harvested from the olfactory bulb. *J. Vet. Med. Sci.* 73 133–137. 10.1292/jvms.10-0344 21293078

[B132] Zhang J. Chen H. Duan Z. Chen K. Liu Z. Zhang L. (2017). The effects of co-transplantation of olfactory ensheathing cells and Schwann cells on local inflammation environment in the contused spinal cord of rats. *Mol. Neurobiol.* 54 943–953. 10.1007/s12035-016-9709-5 26790672

[B133] Zhang J. Liu Z. Chen H. Duan Z. Zhang L. Chen L. (2015). Synergic effects of EPI-NCSCs and OECs on the donor cells migration, the expression of neurotrophic factors, and locomotor recovery of contused spinal cord of rats. *J. Mol. Neurosci.* 55 760–769. 10.1007/s12031-014-0416-2 25239519

[B134] Zheng Z. Du X. Zhang K. Wang X. Chen Y. Kuang N. (2017). Olfactory ensheathing cell transplantation inhibits P2X4 receptor overexpression in spinal cord injury rats with neuropathic pain. *Neurosci. Lett.* 651 171–176. 28461136 10.1016/j.neulet.2017.04.060

