# Supplementary material for: Considering the Cellular Composition of Olfactory Ensheathing Cell Transplants for Spinal Cord Injury Repair: A Review of the Literature
Source: Front Cell Neurosci. 2021 Nov 17;15:781489. doi: 10.3389/fncel.2021.781489 (PMC8635789; doi:10.3389/fncel.2021.781489)
Supplement: Supplementary file 1 [file Table_1.docx]

| **PubMed ID** | **Author** |
| --- | --- |
| 16799702 | Akiyama et al., 2004 |
| 20196694 | Amemori et al., 2010 |
| 15530871 | Andrews and Stelzner 2004 |
| 18001205 | Andrews and Stelzner 2007 |
| 15929557 | Barakat et al., 2005 |
| 10908188 | Barnett et al., 2000 |
| 18973595 | Bretzner et al., 2008 |
| 20568293 | Bretzner et al., 2010 |
| 14691064 | Cao et al., 2004 |
| 16906542 | Cao et al., 2006 |
| 29228020 | Cawardine et al., 2017 |
| 25333925 | Chen et al., 2014 |
| 29285976 | Collins et al., 2018 |
| 23031825 | Coutts et al., 2013 |
| 16498634 | Deng et al., 2006 |
| 15525765 | Dunning et al., 2004 |
| 16219671 | Feron et al., 2005 |
| 14618101 | Gomez et al., 2003 |
| 23169917 | Granger et al., 2012 |
| 28623671 | Gu et al., 2017 |
| 18511045 | Guest et al., 2008 |
| 24380436 | Ibrahim et al., 2014 |
| 19896908 | Kalincik et al., 2010 |
| 26535658 | Kang et al., 2015 |
| 14561871 | Keyvan-Fouladi et al., 2003 |
| 27277804 | Khankan et al., 2016 |
| 12566281 | Lakatos et al., 2003 |
| 23914898 | Lang et al., 2013 |
| 18551623 | Lankford et al., 2008 |
| 15144877 | Lee et al., 2004 |
| 12574399 | Li et al., 2003 (Y) |
| 15246830 | Li et al., 2004 (Y) |
| 26132822 | Li et al., 2016 (YL) |
| 21306739 | Li et al., 2011 (BC) |
| 22407596 | Li et al., 2012 (BC) |
| 15319002 | Lopez-Vales et al., 2004 |
| 11166728 | Lu et al., 2001^117^ |
| 17065452 | Lu et al., 2006^48^ |
| 23638158 | Mayeur et al., 2013^57^ |
| 30322257 | Muniswami and Tharion 2018^95^ |
| 12177207 | Nash et al., 2002^118^ |
| 20932826 | Novikova et al., 2011^75^ |
| 15453992 | Pearse et al., 2004^119^ |
| 17526000 | Pearse et al., 2007^81^ |
| 12614584 | Plant et al., 2003^43^ |
| 14657003 | Radtke et al., 2004^120^ |
| 15067714 | Ramer et al., 2004^44^ |
| 8033963 | Ramon-Cueto and Nieto-Sampedro 1994^64^ |
| 9570810 | Ramon-Cueto et al., 1998^46^ |
| 15185394 | Riddell et al., 2004^121^ |
| 15456822 | Sasaki et al., 2004^123^ |
| 16467529 | Sasaki et al., 2006^122^ |
| 11746771 | Smith et al., 2001^125^ |
| 12359182 | Smith et al., 2002^124^ |
| 20633558 | Stamegna et al., 2011^73^ |
| 24007776 | Tabakow et al., 2013^34^ |
| 25338642 | Tabakow et al., 2014^35^ |
| 21411671 | Takeoka et al., 2011^126^ |
| 17438017 | Toft et al., 2007^79^ |
| 23322541 | Toft et al., 2013^65^ |
| 24146830 | Torres-Espin et al., 2013^127^ |
| 24635194 | Torres-Espin et al., 2014^128^ |
| 12673833 | Verdu et al., 2003^129^ |
| 20447345 | Wang et al., 2010^130^ |
| 20643129 | Wu et al., 2011^131^ |
| 19782053 | Yamamoto et al., 2009 |
| 22551686 | Yazdani et al., 2012^132^ |
| 25239519 | Zhang et al., 2015^134^ |
| 26790672 | Zhang et al., 2017^133^ |
| 28461136 | Zheng et al., 2017^71^ |
|  |  |

**TABLE S1 | Studies of OEC transplantation in SCI repair included in the analysis of the current review.**
