# Supplementary material for: Considering the Cellular Composition of Olfactory Ensheathing Cell Transplants for Spinal Cord Injury Repair: A Review of the Literature
Source: Front Cell Neurosci. 2021 Nov 17;15:781489. doi: 10.3389/fncel.2021.781489 (PMC8635789; doi:10.3389/fncel.2021.781489)
Supplement: Supplementary file 2 [file Table_2.pdf]

Reference	OEC source	Species/ age	Cell culture conditions	Purification method	OECs	Fibroblasts	Meningeal cells	Astrocytes	Endothelial cells	Macrophage/ microglia	Oligodendrocyte progenitor	Oligodendrocyte precursor	Schwann cells	Stem/ neuronal cells/ connective tissue	Unidentified
Rat															
**Barakat et al., 2005**	OB	Rat Adult	FBS, Fsk, PE	$p75^{NTR}$ immunopanning	~96% p75+	"Small number" (no details of marker)							"A few" (no details of marker)		
**Coutts et al., 2013**	OB	Rat Embryonic	FBS, BPE, Fsk	$p75^{NTR}$ immunopanning	>90% p75+/S100+/ GFAP+	<1 – 3% Thy1.1+/Fn+/ Calponin+		<1% p75-/GFAP+							
		Rat Neonatal	FBS, BPE, Fsk	$p75^{NTR}$ immunopanning	>90% p75+/S100+/ GFAP+	<1% Thy1.1+/Fn+/ Calponin+		<1% p75-/GFAP+							
		Rat Adult	FBS, BPE, Fsk	$p75^{NTR}$ immunopanning	>90% p75+/S100+/ GFAP+	<1 – 5% Thy1.1+/Fn+/ Calponin+		<2% p75-/GFAP+							
**Kalincik et al., 2010**	OM	Rat Adult	FBS for 2 days, serum free thereafter	NT3	>90% p75+/GFAP+								<5% non-myelinating SCs (HNK-1)		
**Lakatos et al., 2003**	OB	Rat 7 day pup	FBS, Hrg, Fsk	Unpurified	~60% p75+/ GFAP+		~15% Fn+	~3% GFAP+			~10% A2B5/O4				
				AraC and $p75^{NTR}$ immunopanning	~94% p75+/ GFAP+		"A few" Fn+								
				AraC and $p75^{NTR}$ immunopanning	~70% p75+/ GFAP+		~30% Fn+								
				AraC and $p75^{NTR}$ immunopanning	~50% p75+/ GFAP+		~50% Fn+								
**Lankford et al., 2008**	OB	Rat 4-8 weeks	FCS	Differential adhesion	>95% p75+/GFAP+	<0.1-2% Thy1.1+/ Fn+				<0.1-3% Mac1+ or OX-42+	~0-0.02% NG2+	<0.1-2% 01+			
**Lee et al., 2004**	OB	Rat Adult	FBS	Differential adhesion	50-70% p75+	30-50% (no details of marker)		30-50% (no details of marker)							
**Stamegna et al., 2011**	OM	Rat Adult	FBS for 2 days, serum free thereafter	NT3	>90% p75+/GFAP+								0% myelinating SCs (HNK-1)		
**Toft et al., 2007**	OB	Rat Adult	FBS	Unpurified	~30% p75+	"Varying amounts" Fn+	"Varying amounts" Laminin+		"Varying amounts" SMA+						
		Rat 7 day pup	FCS, FGF2, Hrg, Fsk, ACM	FACS	~98% p75+									~2% Thy1.1+	
**Verdú et al., 2003**	OB	Rat Adult	FCS	Magnetic bead immuno-purification	>97% p75+					"A few" (no details of marker)					
Canine															
**Granger et al., 2012**	OM	Dog 5.9 (+/- 2.0) years	FBS, Fsk, Nrg	Unpurified	~50% p75+	~45% Fn+									~2%
**Smith et al., 2002**	OB	Dog 6 month pup	FBS, Hrg, Fsk	Purified (see paper for details)	~92% p75+/ GFAP+/ O4+	0% Thy1.1+	"Small number" Fn+	<1% GFAP+/ p75-	"Small number" VWF+						
Human															
**Barnett et al., 2000**	OB	Human Adult (15 – 66 years)	FCS	Purified	~30 - 45% p75+	"Some" Fn+	"Some"		"Some" VWF+						
**Collins et al., 2018**	OB	Human		Unpurified	>90% P75+	Very few Fn+									"Other cell types such as mesenchymal stem cells"

TABLE S2 | Details of the characterization of cellular constituents in olfactory cell transplants. A total of 13 studies (19 experiments) provided details of the characterization cells in addition to OECs and fibroblasts in olfactory derived cell transplants. Where values of proportions and details of markers are reported in the publication, these have been detailed in the table. Abbreviations: ACM, astrocyte conditioned media; FBS, fetal bovine serum; FCS, fetal calf serum; FGF, fibroblast growth factor; Fn, fibrofectin; Fsk, forskolin; GFAP, glial fibrillary acidic protein; Hrg, heregulin; Nrg, neuregulin; NT3 neurotrophin 3; p75, $p75^{NTR}$; PE, pituitary extract; SCs; Schwann cells; SMA, smooth muscle actin;VWF, Von Willebrand factor.
